# Neurogenomic divergence during speciation by reinforcement of mating behaviors in chorus frogs (*Pseudacris*)

**DOI:** 10.1186/s12864-021-07995-3

**Published:** 2021-10-02

**Authors:** Oscar E. Ospina, Alan R. Lemmon, Mysia Dye, Christopher Zdyrski, Sean Holland, Daniel Stribling, Michelle L. Kortyna, Emily Moriarty Lemmon

**Affiliations:** 1 Department of Biomedical Sciences, College of Veterinary Medicine, Iowa State University, 1800 Christensen Drive, 50011 Ames, IA USA; 2 Present address: Department of Biostatistics and Bioinformatics, Moffitt Cancer Center, 13131 USF Magnolia Drive, Tampa, FL 33612 USA; 3 Department of Scientific Computing, Florida State University, 400 Dirac Science Library, Tallahassee, FL 32306 USA; 4 Present address: Genetics and Genomics Program, Iowa State University, 2437 Pammel Drive, Ames, IA 50011 USA; 5 Present address: Department of Molecular Genetics and Microbiology, Genetics Institute, University of Florida, Gainesville, FL 32610 USA

**Keywords:** Cascade reinforcement, Behavioral evolution, Mate choice, Transcriptomics, Neurotransmission, Synaptic transmission, Weighted correlation networks, Gene modules

## Abstract

**Background:**

Species interactions can promote mating behavior divergence, particularly when these interactions are costly due to maladaptive hybridization. Selection against hybridization can indirectly cause evolution of reproductive isolation within species, a process termed cascade reinforcement. This process can drive incipient speciation by generating divergent selection pressures among populations that interact with different species assemblages. Theoretical and empirical studies indicate that divergent selection on gene expression networks has the potential to increase reproductive isolation among populations. After identifying candidate synaptic transmission genes derived from neurophysiological studies in anurans, we test for divergence of gene expression in a system undergoing cascade reinforcement, the Upland Chorus Frog (*Pseudacris feriarum*).

**Results:**

Our analyses identified seven candidate synaptic transmission genes that have diverged between ancestral and reinforced populations of *P. feriarum*, including five that encode synaptic vesicle proteins. Our gene correlation network analyses revealed four genetic modules that have diverged between these populations, two possessing a significant concentration of neurotransmission enrichment terms: one for synaptic membrane components and the other for metabolism of the neurotransmitter nitric oxide. We also ascertained that a greater number of genes have diverged in expression by geography than by sex. Moreover, we found that more genes have diverged within females as compared to males between populations. Conversely, we observed no difference in the number of differentially-expressed genes within the ancestral compared to the reinforced population between the sexes.

**Conclusions:**

This work is consistent with the idea that divergent selection on mating behaviors via cascade reinforcement contributed to evolution of gene expression in *P. feriarum*. Although our study design does not allow us to fully rule out the influence of environment and demography, the fact that more genes diverged in females than males points to a role for cascade reinforcement. Our discoveries of divergent candidate genes and gene networks related to neurotransmission support the idea that neural mechanisms of acoustic mating behaviors have diverged between populations, and agree with previous neurophysiological studies in frogs. Increasing support for this hypothesis, however, will require additional experiments under common garden conditions. Our work points to the importance of future replicated and tissue-specific studies to elucidate the relative contribution of gene expression divergence to the evolution of reproductive isolation during incipient speciation.

**Supplementary Information:**

The online version contains supplementary material available at 10.1186/s12864-021-07995-3.

## Background

Species interactions can lead to the divergence of reproductive behaviors, thus reducing the probability of costly hybridization, a process termed reinforcement [1–4]. Furthermore, as interacting taxa diverge, populations within a hybrid zone (sympatry) may incidentally diverge from populations in other hybrid zones or outside of the hybrid zone (allopatry), leading to reproductive isolation within species [3, 5–7]. Theoretical models indicate that the latter process, cascade reinforcement [8], can act as a species multiplier, increasing the rate of speciation within a clade [9–11]. Specifically, as additional taxa arise, novel and spatially-varying species assemblages are formed leading to different selection pressures on reproductive behaviors. The strength of the selection depends upon the combination of closely-related species present in each geographic area [12]. These varying selection pressures may thus contribute to the rapid proliferation of new species [13]. This process has been documented to drive divergence across a broad spectrum of taxa, including fish [14], frogs [15–17], *Drosophila* [18–23], walking-stick insects [24], beetles [25], and flowering plants [26, 27].

Systems experiencing cascade reinforcement provide a unique opportunity to study the genetic changes that occur during the first stages of speciation [28]. Although little is known about the genetic targets of selection underlying in this mechanism, some information is available from studies of the more general process of reinforcement between species [26, 29–31]. For example, previous work has identified signatures of selection, such as selective sweeps, at documented [32] and candidate [33, 34] reinforcement loci, under the assumption that loci experiencing strong selection in sympatry should show decreased genetic variation [28].

As predicted by theory [35], the evolution of reproductive isolation during reinforcement can result from structural changes at the genomic level [36, 37]. Diverging species and populations are also likely to undergo differentiation of gene regulation pathways [38, 39]. Because gene expression is regulated through complex interactions of sequences and proteins within the cell, these interacting regulatory elements are thought to be co-adapted [40]. When these co-adapted complexes are disrupted, such as in hybrids, the regulatory targets may be mis-expressed, resulting in incompatibilities between species or divergent populations [39, 41]. These incompatibilities are particularly likely to contribute to reproductive isolation when selection targets different components of a gene network [42], which can occur in replicate sympatric populations during cascade reinforcement.

In the well-studied European house mouse system (*Mus musculus* spp.), Loire et al. [34] identified candidate mate recognition genes by studying gene expression divergence during reinforcement. Specifically, they found differential gene expression of candidate vomeronasal receptor genes between populations of the same subspecies within and outside of a reinforced contact zone. Loire et al. [34] expected to find divergence of these candidate genes because behavioral studies indicated that mice use pheromone-based information in urine for discriminating between subspecies [43–47]. Their findings suggest that divergence of olfactory gene expression is also contributing to the evolution of assortative mating among populations within one of the subspecies [47]. In the only study of gene expression in a frog system undergoing reinforcement, the spadefoot toads (*Spea* sp.), Seidl et al. [41] determined that divergence of gene expression profiles accompanies divergence of reproductive behaviors in sympatry. In spadefoots, the interacting species have evolved increased species discrimination within their contact zone, due to the negative consequences of hybridization [48, 49] (but see [50]). Seidl et al. [41] found that hybrids derived from interspecific crosses of parents originating in sympatry differed significantly in gene expression from their counterparts generated from allopatric parents. They concluded that ongoing hybridization in sympatry is driving the divergence of gene expression between geographic regions. These empirical studies support theoretical predictions that reinforcing selection can contribute to divergence of gene expression across populations, pointing to the need for studies on additional non-model systems.

**Fig. 1:**
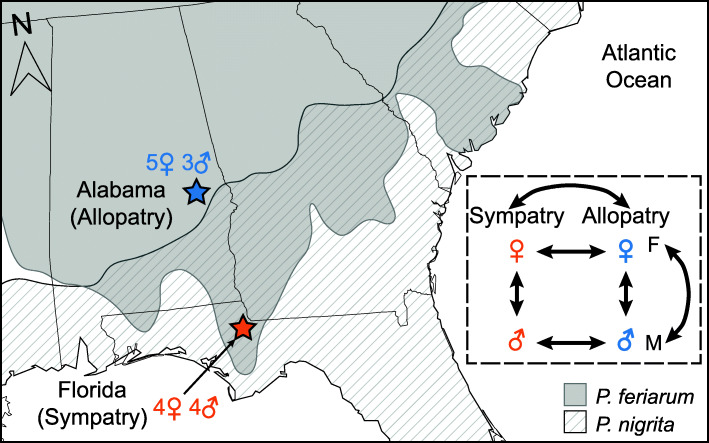
Geographic locations of the collected specimens in this study within southeastern United States. The gray area indicates the distribution of *P. feriarum*, whereas the area with diagonal lines represents that of *P. nigrita*. Allopatric frogs (red star) were collected in Macon County, Alabama. Sympatric frogs were collected in Liberty County, Florida (blue star). The number of specimens from each sex is shown. The inset dashed box shows all the comparisons performed to detect differentially expressed genes. This map was generated by the authors using QGIS v3.1

In the North American chorus frogs (genus *Pseudacris*), reinforcement in contact zones has caused one species (*P. feriarum*) to diverge in male acoustic signals and female mating preferences from another species (*P. nigrita* [51]). As a consequence, increased reproductive isolation has evolved between the two species in sympatry [51, 52]. The lineages giving rise to *Pseudacris feriarum* and *P. nigrita* diverged ~ 8 million years ago (ma) based on estimates from mitochondrial markers, and the two taxa have come into secondary contact more recently [53, 54]. Phylogeographic evidence indicates that *P. feriarum* expanded into the range of *P. nigrita* multiple times, by following the floodplains of river systems that bisect the Coastal Plain of the southeastern United States (Fig. 1 [55]). Thus, allopatric *P. feriarum* represent the ancestral state for the species, whereas sympatric *P. feriarum* represent the derived, reinforced state. Empirical work indicates that populations within *P. feriarum* are diverging via cascade reinforcement: sympatric *P. feriarum* females have evolved strong preferences for sympatric over allopatric male signals, and male calls have diverged substantially between regions [51]. Estimates of the timing of secondary contact with *P. nigrita* are uncertain; mitochondrial-based estimates range from 0 to 4 ma [56]. Within *P. feriarum*, genetic divergence between allopatric and sympatric (with *P. nigrita*) regions including the populations in this study are low (F_ST_ = 0.04), and less than between adjacent allopatric regions of similar geographic distance (F_ST_ = 0.12 [55]). The significant differentiation of both male and female reproductive behaviors between ancestral and reinforced populations suggests that the sexual decision-making genetic pathways have diverged in the brains of sympatric frogs [51]. Furthermore, because the cost of hybridization is higher for females than males, female *P. feriarum* may have diverged to a greater degree than males, due to strong natural and sexual selection on female mate preferences [52].

Reinforcement selection may have driven divergence in expression of genes involved with mating behaviors, particularly within the auditory midbrain. Neuroethological studies in another chorus frog species (*P. regilla*), have revealed that critical elements of male acoustic signals are decoded in the inferior colliculus of the auditory midbrain [57, 58]. Electrophysiological analyses have identified two classes of pulse-rate selective neurons in this brain region: interval-counting neurons (ICNs) and long-interval neurons (LINs) [59–63]. ICNs show band-pass selectivity for pulse rate and respond only after a threshold number of pulses are presented with optimal interpulse onset intervals [59]. Importantly, these two acoustic characteristics  of mating signals diverged between allopatric and sympatric populations of *P. feriarum* [51]. The tuning of ICNs for pulse rate and interval counting involves interplay between excitation via AMPA-type and NMDA-type ionotropic glutamate receptors and inhibition via ionotropic GABA_A_ receptors [61, 64]. Our preliminary electrophysiological data suggest that allopatric and sympatric *P. feriarum* differ with respect to the tuning of ICNs in the auditory midbrain (E. Lemmon and G. Rose, unpublished observations). These differences may be mediated by divergence in genes and/or their expression that govern the presence and function of inhibitory and excitatory synapses. For example, up-regulation of one or more genes that encode GABAergic receptors in ICNs may increase the relative amplitude of inhibition and as a consequence increase the number of pulses required for eliciting responses [64, 65] (G. Rose, unpublished observations). Thus, changes in expression of ionotropic receptor genes and other genes related to synaptic transmission might underlie the observed differences in mating behaviors between ancestral and reinforced populations.

In this study, we aim to better understand the changes that occur in the earliest stages of speciation via cascade reinforcement in *P. feriarum*. Examining populations of the same species that are experiencing different selection pressures on their reproductive behaviors allows us to focus more directly on early genetic changes as speciation proceeds [28]. To this aim, we generate a comprehensive reference transcriptome for *P. feriarum* using a variety of tissues, then use a hypothesis-testing approach to narrow down a large set of candidate genes involved in synaptic transmission in the brain to a smaller set that have diverged between ancestral and reinforced populations. We predict that one or more genes involved in synaptic transmission will be differentially expressed between ancestral and reinforced populations in one or both sexes.

In order to understand the general patterns of divergence across the entire transcriptome, we also study expression changes (between sexes and between populations) for the entire set of genes expressed in the brain. We make several predictions based on our current understanding of the neurophysiology and evolutionary history of these frogs. First, we expect that gene expression patterns have diverged to a greater degree between ancestral and reinforced populations than between the sexes, due to the effects of natural and sexual selection via reinforcement [52]. Second, we predict that females from ancestral and reinforced populations will show the greatest divergence, since females experience a greater cost to hybridization and thus stronger selection than males. If the first prediction is upheld, the pattern could alternatively be explained by differences in environmental selection (i.e., abiotic factors, such as climate, or other biotic factors, such predator communities), or by historical demographic differences between populations. However, if the second prediction is also upheld, these two alternative hypotheses would be less likely because the sexes should be affected similarly by environmental factors or demographic shifts. Third, we predict that genes involved in synaptic transmission will be overrepresented in one or more gene networks that have diverged between ancestral and reinforced populations, since neural processing by auditory neurons of the midbrain are thought to mediate mate choice in this system.

## Results

### Reference transcriptome assembly

We obtained a comprehensive reference transcriptome. Before filtering and processing, we obtained between 28 million and 56 million read pairs from each tissue (brain, eye, testis, liver, heart, lung, skin, and leg muscle) from two individuals. In all cases, 98% of the reads had high average quality with Phred score > 28 and average length of 100 bp (Additional file 1: Table S1). Following trimming and rRNA filtering our transcriptome data set contained between 27 million and 55 million read pairs per tissue type with 99% of the sequences showing Phred score > 28. After assembly, we obtained 598,748 contigs representing 387,298 unique transcripts with average, median and N50 length of 639,325, and 1019 bp respectively. This set of transcripts was considered as our assembled reference transcriptome for all downstream analyses. A different set of sequences (*Results—Brain Expression Transcriptomes*) was mapped to the reference transcriptome to study gene expression. The BUSCO (Benchmarking Universal Single-Copy Orthologs) search for ortholog genes in the Tetrapod ortholog database showed a coverage of 95.2% for the assembled transcriptome, representing 3761 complete single-copy orthologs. The mean and median length of the contigs matching subjects in BUSCO was 2669 and 2272 bp, respectively, meaning that contigs larger than the whole transcriptome median contig length (~ 360 bp) contributed to ortholog detection. Length coverage analyses to assess protein length (based on blast searches; *Methods—Reference Transcriptome Assembly*) showed that 10,993 of the UniProt/SwissProt and 13,779 proteins of the *Xenopus tropicalis* databases matched more than 90% of their aminoacidic lengths to transcripts in our assembled transcriptome (Additional file 1: Table S2).

The annotation (performed in Trinotate; *Methods—Reference Transcriptome Assembly*) based on the UniProt/SwissProt database yielded identification for 99,774 (26%) of the transcripts. Of those, 18,358 transcripts were associated with *Xenopus tropicalis*. For the UniProt/SwissProt annotations, our reference transcripts matched with 21,841 Gene Ontologies (GOs). Annotation using the Pfam database for protein families yielded 48,469 annotations (12.5% of transcripts), of which 2283 were associated with GO terms. Overall, 11,512 different GO terms were associated with biological processes, 3995 with molecular functions, and 1751with cellular components (Additional file 2: Fig. S1).

### Brain transcriptomes

We quantified gene expression in 17 individual *P. feriarum* from four groups: sympatric females (*n* = 5), sympatric males (*n* = 4), allopatric females (*n* = 5), and allopatric males (*n* = 3). After processing and filtering, the number of paired reads from each sample ranged from 14.9 to 97.6 million sequences (Additional file 1: Table S3). Of the 17 samples sequenced, all but one produced data of sufficiently high quality to be included in downstream analyses (Additional file 3: Fig. S2); one sympatric female sample produced substantially fewer post-filter read pairs (14.9 million) than the remainder of the samples (mean = 55 million, min = 34 million). Data for this individual were not carried forward in the downstream analyses presented below. On average, 64% of the reads from each sample mapped to our reference transcriptome (61–69%). Studies mapping expression reads to a more comprehensive, pre-existing transcriptome have achieved similar mapping percentages (e.g., [66]). None of the 16 samples appeared as outliers in the multidimensional scaling (MDS) analysis of the Trimmed Mean of M-values (TMM) log2 counts per million (CPM) values (Additional file 4: Fig. S3).

### Identification of candidate synaptic transmission genes

From published studies [61, 64, 65, 67, 68], we identified 39 target protein families associated with synaptic transmission (Table 1; *Methods—Identification of Candidate Gene Transcripts Involved in Synaptic Transmission*). We obtained matches to 97% (38 of 39) of these target protein families in the expression transcriptome: 1104 (~ 2%) of reference transcripts contained one or more keywords. The one exception is, Pp60src, a tyrosine kinase that phosphorylates synaptophysin and synaptogyrin. The keywords matching this target gene were not found in the annotation terms of any of the transcripts in the reference transcriptome. Of the matching reference transcripts, 554 were present in the expression data set (i.e., passed the quality/coverage filters). These transcripts, which we hereafter refer to as the candidate synaptic transmission genes, included 54 ionotropic receptors, 259 synaptic vesicle proteins, 126 proteins that associate with synaptic vesicles, 99 synaptic plasma membrane proteins, and 16 proteins that reversibly associate with plasma membrane proteins [67].

**Table 1:** Categories of predicted differentially-expressed genes between allopatric and sympatric populations. Categories and genes involved in synaptic transmission for the anuran auditory pathway are summarized from [67, 68]. Gene and protein names indicate significant differentially-expressed transcripts between allopatric and sympatric populations for each gene/gene family after FDR correction (0.05)

Categories of Predicted Proteins	Diff. Expr. Gene Name	Diff. Expr. Protein Name
1. **Ionotropic receptors**		
Glutamate receptor ionotropic, NMDA	–	–
Glutamate receptor ionotropic, AMPA	–	–
Gamma-aminobutyric acid receptor (GABA)	–	–
2. **Synaptic vesicle proteins**		
Neurotransmitter transporters	Slc6a12; BGT1	Sodium- and chloride-dependent betaine transporter /GABA transporter
Neurotransmitter transporters	Slc6a13; GAT2	Sodium- and chloride-dependent GABA transporter 2
Cysteine string protein (CSP)	–	–
Cytochrome b561	–	–
Rab and Ra1 proteins	–	–
Rabphilin-3A	–	
Secretory carrier membrane proteins (SCAMPs)	SCAMP3	Secretory carrier-associated membrane protein 3
Synaptic vesicle protein (SV2)	–	–
Synapsins	–	–
Synaptobrevins	–	–
Synaptogyrin	–	–
Synaptophysin	–	–
Synaptotagmin	ESYT2-b	Extended synaptotagmin-2-B
Transport proteins (channels) for chloride and zinc^a^	SLC39A10	Zinc transporter ZIP10
Vacuolar proton pump	–	–
3. **Proteins that associate with synaptic vesicles**		
Amphiphysin	–	–
Endophilin	–	–
AP2 and clathrin	–	–
Ca2+, calmodulin-dependent protein kinases I and II (CaMKI and CaMKII)	–	–
Dynamin-1	–	–
Dynein	–	–
Kinesins	KIFA3	Kinesin-associated protein 3
Guanine nucleotide exchange factor MSS4	–	–
Pp60src (tyrosine kinase that phosphorylates synaptophysin and synaptogyrin)	–	–
Complexin/synaphin		
4. **Synaptic plasma membrane proteins**		
Munc13s	–	–
Neurexins	–	–
SNAP-25	–	–
Syntaxins	–	–
Voltage-gated Ca2+ channels	–	–
RIM	–	–
Neuroligin		
Cadherin	CDH15; M-cadherin	Cadherin-15
5. **Proteins that reversibly associate with plasma membrane proteins**		
Munc18s/syntaxin binding protein	–	–
N-ethylmaleimide-sensitive factor (NSF)	–	–
α/β/γ SNAPs	–	–

^a^Members of the ZnT zinc transporter family are from Category 2, but those from the ZIP zinc transporter family, which occur in the plasma membrane instead, are from Category 4 [94]s

### Differential expression of candidate synaptic transmission genes

**Fig. 2:**
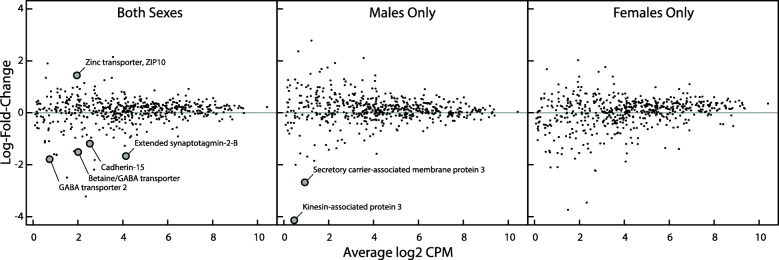
Differential expression of candidate synaptic transmission genes. Each point represents one of the 554 candidate transcripts for which expression data were compared between ancestral (allopatric) and reinforced (sympatric) populations. Comparisons were made using all samples (left graph), males only (center graph) and females only (right graph). Effect size (log2 fold change) is plotted against average expression (log2 counts per million). Genes identified as significant at 0.05 level (after FDR correction) are denoted by larger gray points

Analysis of the candidate synaptic transmission genes revealed seven genes with significant expression differences by geography between populations, two in males only and five when both sexes were combined (Additional file 1: Table S4). The log difference ratios of these genes are highlighted in Fig. 2. Five of the differentially-expressed (DE) genes are synaptic vesicle proteins: Secretory carrier-associated membrane protein 3 (SCAMP3, False-Discovery Rate [FDR] = 0.5 × 10^-3), Synaptotagmin-2-B (ESYT2-b, FDR = 0.038), GABA transporter 2 (Slc6a13, FDR = 0.038), Sodium and chloride-dependent betaine/GABA transporter (Slc6a12, FDR = 0.038), and Zinc transporter ZIP10 (Slc39a10, FDR = 0.038). The two remaining differentially-expressed genes include a protein that associates with synaptic vesicles, kinesin-associated protein 3 (KIFA3, FDR = 4.87 × 10^-6), and a synaptic plasma membrane protein, cadherin-15 (CDH15, FDR = 0.038). Note that sampling 554 genes (candidate synaptic transmission genes) at random from the entire set of genes results in 8.4 significant genes on average, suggesting that the synaptic transmission gene candidate set does not contain a greater number of differentially expressed genes than would be expected [P (number significant) > 6 = 0.634], given the size of the gene set.

### Comparing expression divergence among the four groups

Expression trees showing the relative divergence among the four groups (Additional file 5: Fig. S4; Additional file 1: Table S5) did not identify a group in which expression divergence was greatest (all FDR were greater than 0.08). Moreover, when comparing the ratio of branch lengths (representing the relative amount of evolution of two groups) between the candidate genes and non-candidate genes, we found no difference (all FDR were greater than 0.08), suggesting that the relative rates of evolution for the four groups were not enhanced in the candidate gene set.

### Transcriptome-wide patterns of divergence

**Fig. 3:**
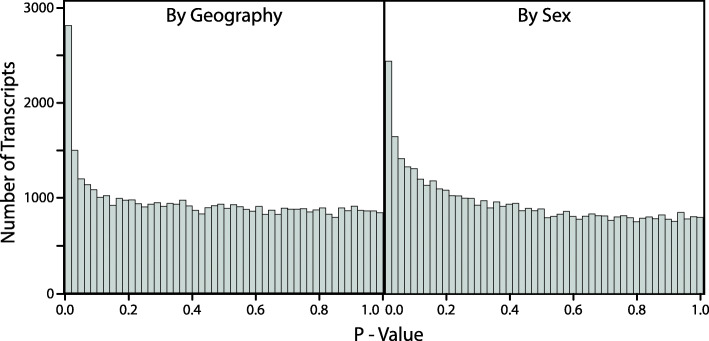
*P*-value distributions. Likelihood ratio tests were used to test for differential expression between ancestral (allopatric) and reinforced (sympatric) populations (left graph) and between females and males (right graph). The increased number of transcripts in the left tail of the distributions indicate the presence of genes that are significantly differentially expressed

**Fig. 4:**
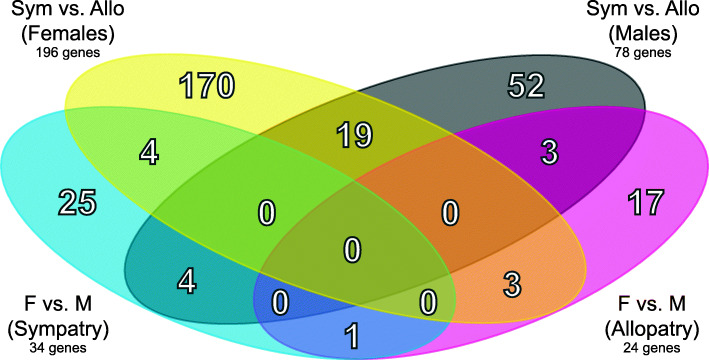
The number of differentially expressed genes among comparisons of *P. feriarum* brains (FDR < 0.05). Each comparison is depicted as an oval and the category in parentheses indicates the data subset in which the comparison was made. In total 298 differentially expressed genes were detected. Note that the highest number of differentially expressed genes (*n* = 196) was found between sympatric and allopatric females

**Fig. 5:**
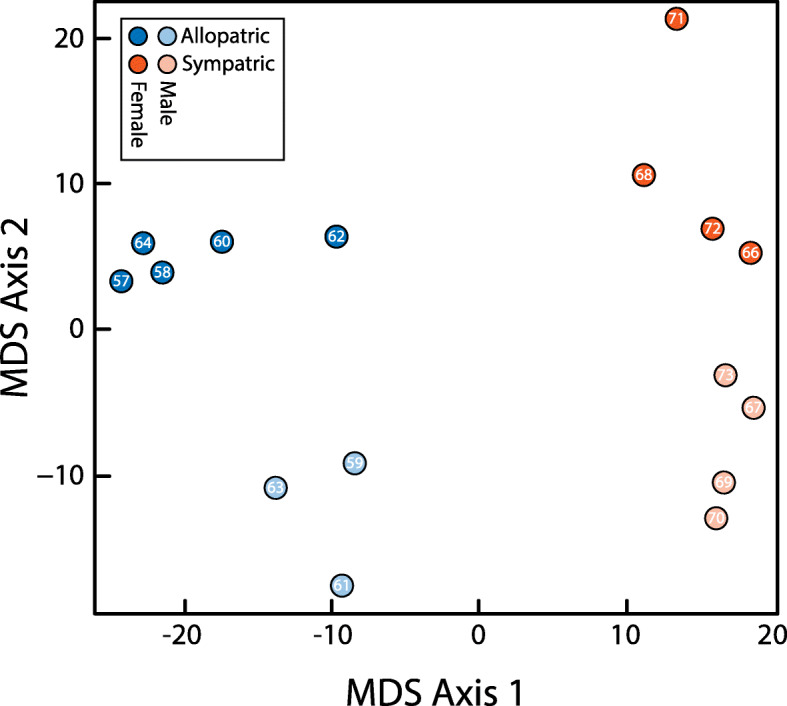
Multidimensional Scaling Plot of pairwise expression differences, based on normalized log2 counts per million for the differentially expressed genes in the transcriptome-wide gene set. Note the discrete break between the two populations (allopatric and sympatric). Also note the higher level of dispersion for sympatric samples than allopatric samples. Numbers inside points correspond to the last two digits of the sample IDs in Additional file 1: Table S10

Our transcriptome-wide analysis, based on 48,254 genes, (*Methods—Evaluating Transcriptome-wide Patterns of Divergence*), revealed a substantial number of differentially-expressed genes (Fig. 3), with the greatest difference in expression seen between ancestral and reinforced populations (Fig. 4). Among the four Geography x Sex comparisons, we detected 298 differentially-expressed genes based on an FDR threshold of 0.05 (Additional file 1: Table. S6). The highest number of differentially-expressed genes detected were between sympatric and allopatric females (*n* = 196), however, a substantial number was also identified between sympatric and allopatric males (*n* = 78), 19 of which were shared between these two comparisons. A multidimensional scaling (MDS) plot based on pairwise log2-fold changes of all DE genes separated the samples primarily by geography (sympatric versus allopatric populations) and to a lesser degree by sex (Fig. 5).

The majority of differentially-expressed genes were observed between populations. The results from the LRTs (Likelihood ratio tests) showed 517 differentially-expressed genes between all allopatric frogs and all sympatric frogs. Note that with the FDR correction and an alpha threshold of 0.05, there is only a 5% chance of one or more genes showing significant differential expression. Of the genes differentially expressed by geography, 166 were overexpressed in sympatry and 351 were underexpressed in sympatry (see Additional file 1: Table S6 for effect sizes). The hierarchical clustering analysis supported the separation between the two groups with an approximately unbiased *p*-value (AU, a multiscaled version of bootstrap [69]), AU > 86% (Additional file 6: Fig. S5).

**Fig. 6:**
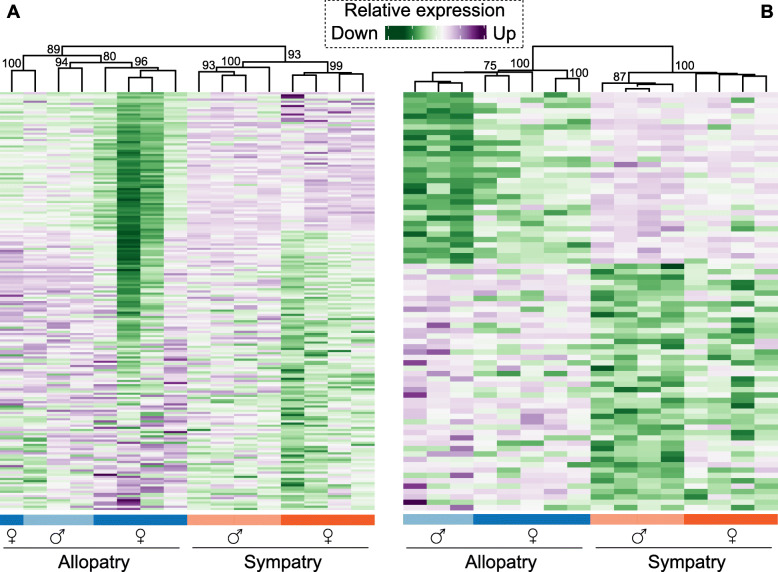
Relative expression levels (log_2_ CPM) across all samples of 196 differentially expressed genes between sympatric and allopatric females (**A**), and 78 differentially expressed genes between sympatric and allopatric males (**B**). The dendrograms resulted from hierarchical clustering of expression levels after 100 replicates to estimate Approximately Unbiased *p*-values (numbers on nodes). Note that the differences between dendrograms require that the individuals and groups be presented in different order at the bottom of the heatmaps

Between allopatric and sympatric females, 196 genes were differentially expressed. A clustering analysis based on those genes provided high support (AU > 89%) for the separation of allopatric females from sympatric females (Fig. 6A). Between allopatric and sympatric males, 78 genes were differentially expressed that separated these two groups (AU = 100%, Fig. 6B). As expected, differentially-expressed genes between females and males allowed statistically significant clustering of transcriptomic profiles based on sex (Additional file 7: Fig. S6). The contrast between brains of all female and all male frogs yielded 129 differentially-expressed genes. Of those, 81 were underexpressed in females and 42 were overexpressed in females. The hierarchical clustering analysis supported a cluster including most females except two from sympatry (AU = 88%; Additional file 7: Fig. S6). The same two females were included in a second cluster with the male samples (AU = 73%). We found 24 differentially-expressed genes between females and males in allopatry. Overall, these differences in gene expression did not form hierarchical clusters as well supported as those observed in the comparison between allopatric and sympatric frogs. Allopatric males and allopatric females formed two clusters supported by AU = 99% (Additional file 8: Fig. S7).

More genes were differentially expressed by geography than by sex, as indicated by our randomization tests (ratio = 4.4737, *p* < 0.0001). The randomization tests also demonstrated that females had more genes differentially expressed by geography than males (ratio = 2.51, *p* < 0.0001). We did not, however, find a significant difference between sympatry and allopatry in the number of genes differentially expressed by sex (ratio = 1.4167, *p* = 0.1217).

### Divergence of brain gene co-expression networks

The analysis of co-expression network yielded 25 modules (31 before merging) of genes with correlated expression levels and high connectivity among them (Additional file 9: Fig. S8; Additional file 1: Table S7-S8). The co-expression networks were constructed from 4826 genes in the top 10% with respect to the amount of among-sample variation in normalized CPM. The network filtered at correlation coefficients (*r*) lower than *r* = 0.05, contained 2871 transcripts with *r* ≥ 0.1, and 513 transcripts showing connections with *r* ≥ 0.3. The average degree of connectivity (number of connections) in this co-expression network was 325.6, with average clustering coefficient (C) and average neighborhood connectivity (NC) of C = 0.7 and NC = 637.1, respectively, indicating overall high connection among all genes and their neighbors. A total of 372 differentially-expressed genes from any of the previously mentioned comparisons were included in this network analysis.

**Fig. 7:**
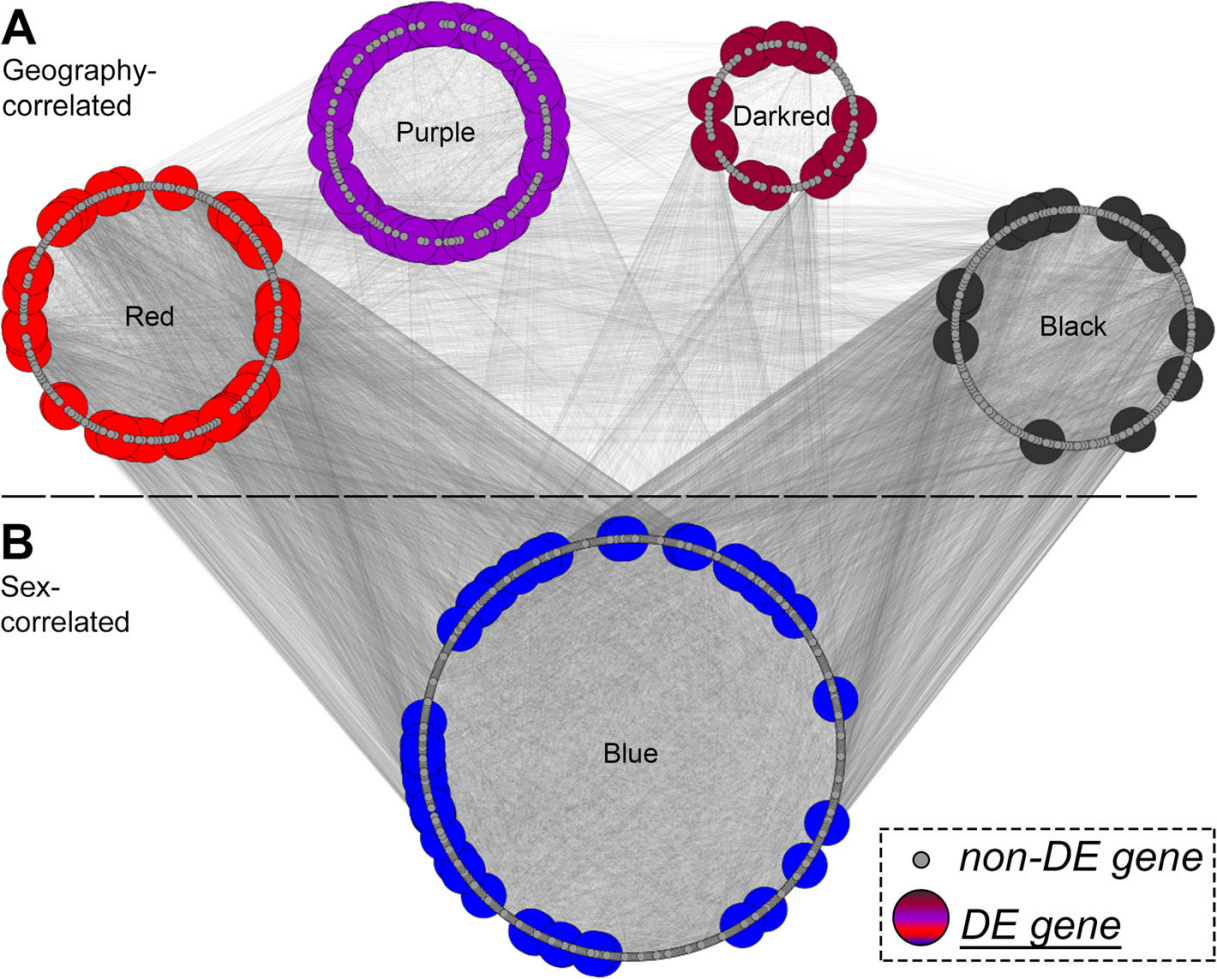
Representation of modules in the brain co-expression network that have significant association with **A** geography (sympatry/allopatry) or **B** sex. Each node (circles) represents a gene, with larger colored nodes indicating DE genes resulting from any of the comparisons conducted across all groups. The density of thin grey lines between the modules provides a visual representation of the connectivity within the co-expression network

Correlation tests yielded four modules with significant association between overall gene expression and geography (Fig. 7; Fig. S8; Additional file 1: Table S8-S9). Those modules were named darkred, purple, red, and black following WGCNA’s module notation. One of those modules, named darkred, included 71 genes that showed expression levels associated with geography (*r* = 0.65, FDR = 0.0260). This module showed average connection degree of 9.0, C = 0.6, and NC = 13.7. The darkred module contained 14 differentially-expressed genes. A second significant module, termed the purple module, included 156 genes that exhibited expression levels associated with geography (*r* = 0.93, FDR < 0.0001). The purple module showed an average degree of connectivity of 15.4 and C = 0.6, and NC = 28.9 and contained 63 differentially-expressed genes. A third module, named red, contained 196 genes showing association of expression levels with geography (*r* = − 0.8, FDR = 0.0019). The average degree of genes within this module was 41.6, C = 0.8, and NC = 81.3. The red module contained 39 differentially-expressed genes. The fourth module, which was called the black module, contained 190 genes with gene expression patterns correlated with geography (*r* = 0.6, *p* = 0.0260). The genes in this module had an average degree of 39.6, C = 0.76, and NC = 68.2. The module included 14 differentially-expressed genes.

Only the blue module had gene expression associated with sex (Fig. 7; Additional file 9: Fig. S8; Additional file 1: Table S8-S9). The blue module included 575 genes with expression levels correlated with a frog’s sex (*r* = 0.64, *p* < 0.0260). The genes in this module showed an average degree of 279.2, C = 0.9, and NC = 381.0. Of the 575 genes in the blue module, 44 were differentially expressed.

The 36 neurotransmission-related enriched GO pathways identified (Additional file 1: Table S9) were not randomly distributed across the modules but are instead significantly concentrated in the darkred module (15 of 28; FDR < 0.0001), the purple module (9 of 27, FDR = 0.0088), and the white module (11 of 40; FDR = 0.0125; note the white module was not significantly correlated with geography or sex; Fig. S8; Table S8-S9). Moreover, the modules associated with geography contained significantly more synaptic pathways than modules not associated with geography (randomization test, ratio = 3.40, *p* < 0.0001).

## Discussion

Our study has revealed multiple candidate synaptic genes and four gene modules that have diverged between ancestral (allopatric) and reinforced (sympatric) populations of *P. feriarum*. Two of these modules are significantly enriched for synaptic transmission functions. We found a greater number of genes have diverged in expression between ancestral and reinforced populations than between the sexes. Furthermore, we observed a significantly greater number of genes that are differentially expressed by population in females compared to males. In contrast, we found no difference in the number of genes differentially expressed by sex within the ancestral compared to the reinforced population. Although this study represents just the first glimpse into brain gene expression divergence during cascade reinforcement in this system, our results are consistent with neurophysiological and behavioral studies suggesting that changes in synaptic transmission may lead to divergence of mate recognition mechanisms during speciation.

### Transcriptome-wide divergence of ancestral and reinforced populations

Our data are consistent with the idea that natural and sexual selection driving reinforcement in this system have contributed to differential gene expression between ancestral and reinforced populations. Support for this interpretation comes from the patterns of greater gene expression divergence by geography than by sex and of greater divergence by geography in females than males. Together, these patterns suggest that environmental selection and demographic processes may not be the only factors contributing to divergence by geography, but rather, stronger selection against hybridization in females than males may also underlie these differences [51, 52].

Some of the observed gene expression divergence by geography may be due to factors other than reinforcement, such as environmental selection or demographic history of the populations. For example, in terms of environmental differences, sympatric *P. feriarum* in Florida primarily breed in cypress-gum swamps along river floodplains, whereas allopatric conspecifics in Alabama utilize wetlands in more general mixed temperate forest habitats. These differences suggest that sympatric frogs may be more restricted with regard to their habitat requirements. A previous study of environmental selection on acoustic signals found, however, that sensory drive [70–72] is not driving divergence of these behaviors [73]. This study, which included the two populations in the current study, suggests that habitat selection has not contributed significantly to divergence of acoustic signals between populations. Moreover, overall environmental conditions of the two locations are similar (e.g., Level III Ecoregion [74]).

The two populations examined in our study may also differ somewhat in their demographic histories. Repeated contact zones have formed across *P. feriarum* and *P. nigrita* distributions [55]. In these contact zones, *P. feriarum* has expanded into the range of the latter species multiple independent times by following different river systems of the southeastern U.S. (e.g., Escambia, Apalachicola, Altamaha, Edisto/Santee, and James/Anna Rivers). In each contact zone, migration estimates point to ongoing unidirectional gene flow, from the ancestral allopatric region into the reinforced sympatric areas [55]. Estimates of effective population sizes (N_E_) are equivalent to or larger in replicate sympatric compared to allopatric populations across all five river systems [55], suggesting that that drift is not predicted to be stronger in peripheral sympatric populations. Consistent with the migration data, estimates of genetic divergence between allopatric and sympatric regions including the populations in this study are low (F_ST_ = 0.04), and less than between adjacent allopatric regions of similar geographic distance (F_ST_ = 0.12 [55]).

### Divergence of brain gene co-expression networks

Of the four genetic modules that diverged between the ancestral and reinforced populations, two were enriched for and showed significant concentration of terms related to neurotransmission (darkred FDR < 0.0001 and purple modules FDR = 0.0088) compared to all other network modules. Specifically, darkred module terms included integral and intrinsic components of the presynaptic and postsynaptic membrane, postsynaptic density, and extrinsic component of plasma membrane, among others (Additional file 1: Table S9). These terms encompass the products of the candidate genes that were found to be differentially expressed by geography in this study (see below). Purple module terms involved metabolism of the neurotransmitter nitric oxide (NO). The NO pathway modulates the release of multiple neurotransmitters [75, 76], and has been shown to be involved in mating behaviors [77, 78]. Mate choice studies in *Drosophila* have shown that in neurons of the ellipsoid body of the brain, NO acts as a second messenger after stimulation by GABA via GABA-A receptors to contribute to activation of NMDA receptors, resulting in the alteration of mate decisions by females [79, 80]. This connection between nitric oxide, synaptic transmission via ionotropic receptors, and mate choice is consistent with computational models of how the temporal information present in acoustic signals is processed in the auditory midbrain [81].

Of the other two divergent modules that did not show concentration of neurotransmission terms, one primarily included terms for enzymatic activity (red module), and another for transcription factor binding and citric acid cycle (black; Additional file 1: Table S9). Overall, a variety of synaptic pathways were concentrated in the significant modules (*p* < 0.0001), suggesting that neural pathways are diverging in gene expression between ancestral and reinforced populations. Note that the divergent modules only contained a marginally significant number of synaptic pathways (per module) compared to the non-significant modules (*p* = 0.0561).

Although the other 21 modules did not show significant divergence geographically, one of these was also enriched for neurotransmission-related terms, including inner ear receptor cell development and differentiation, sensory organ development, neurogenesis, and mechanoreceptor differentiation (white). The only sex-associated divergent module was enriched for terms such as transmembrane transporter activity of solutes, hormone activity, and neuroactive ligand-receptor interaction (blue; Additional file 1: Table S9).

### Divergence of candidate synaptic transmission genes

We predicted that gene expression has diverged between ancestral and reinforced populations across five possible categories of proteins involved in synaptic transmission (Table 1). We identified seven candidate genes across three categories, all of which were also present in our transcriptome-wide set of differentially-expressed genes (Additional file 1: Table S4). For the ionotropic receptors (Category 1), we found no evidence of differential gene expression. Because neurophysiological studies of midbrain neurons [57–60, 63–65] indicated that these receptors mediate excitation and inhibition of auditory neurons, we hypothesized that down- or up-regulation of one or more genes that code for these receptors may alter how acoustic information is processed. We did not find any evidence for expression changes in ionotropic receptor genes, however, at the whole-brain level.

With respect to synaptic vesicle proteins (Category 2), we found five genes that were differentially expressed between the ancestral and reinforced populations. Two of these genes were GABA transporters (Sodium- and chloride-dependent GABA transporter [GAT2; Slc6a13] and Sodium- and chloride-dependent betaine transporter/GABA transporter [BGT1; Slc6a12]). The primary function of GABA transporters in the brain is to recycle GABA neurotransmitter from the extracellular space of the synaptic cleft and through the cell membrane into the presynaptic neuron [82]. Although expressed in brain tissue, GAT2 and BGT1, are less common in GABAergic synapses than other GABA transporters (e.g., GAT1 and GAT3). GAT2 and BGT1 are not thought to play a major role in GABA reuptake in neurons compared to GAT1 and GAT3 due to lower expression levels, lower binding affinities to GABA, and localization to other areas of the brain [83–86].

In regard to other synaptic vesicle proteins (Category 2), the other three differentially-expressed genes included Secretory carrier-associated membrane protein 3 (SCAMP3), Extended-synaptotagmin-2-B (ESYT2-b), and Zinc transporter ZIP10 (Slc39A1). The SCAMP3 gene encodes a secretory carrier membrane protein that functions as a carrier to the cell surface in post-golgi recycling pathways [87]. One function of SCAMP proteins is to bind to neurotransmitter transporters (Slc6 genes, see above) and regulate their expression at the cell surface [88, 89]. Although little is known about the role of SCAMPs in behavior, a study in *Drosophila* reported multiple abnormal behaviors in SCAMP-null mutants, underscoring the importance of this gene in neurotransmission [90].

The extended-synaptotagmins (ESYTs) are a class of endoplasmic reticulum (ER)-resident proteins that are related to but predate the synaptotagmin genes evolutionarily [91]. These proteins are thought to coordinate membrane tethering and lipid exchange between the endoplasmic reticulum and plasma membrane. Studies in *Drosophila melanogaster* of the single ESYT ortholog in this species found that neurotransmission is reduced in ESYT mutants, indicating that this gene is required to facilitate presynaptic release of neurotransmitter [92]. Furthermore, synaptic growth is promoted by overexpression of this gene [92].

Zinc transporters of the zinc importer (ZIP or Slc39A) family control the influx of zinc from outside the cell into the cytoplasm and from vesicles to increase cytosolic zinc concentrations [93–95]. Unlike the second family of zinc transporters (ZnT or Slc30), which can be components of synaptic vesicles, ZIP family proteins can be located in the plasma membrane of neurons [95]. Zinc plays a diversity of roles in the brain, influencing neurotransmission and sensory processing and is of particular interest to the current study because its involvement with inhibition of NMDA and GABA-A receptors in the postsynaptic membrane [96–98]. Genome-wide association studies have found a strong association between ZIP zinc transporters and several psychiatric disorders [99, 100], indicating the key role of these transporters for healthy functioning of the central nervous system [97].

In terms of proteins that associate with synaptic vesicles (Category 3), Kinesin-associated protein 3 (KIFAP3 or KAP3) was differentially expressed between ancestral and reinforced populations. Kinesins are anterograde (cell body to the synaptic junctions) motor proteins that traffic vesicles by walking them along microtubules [101, 102]. The KIFAP3 gene encodes a non-motor accessory protein that forms a complex (KIF3 complex) with two kinesin 2 family proteins to generate a functional motor. This complex provides essential transport of vesicles during axonal elongation, conventional transport, and flagellar transport [103]. The KIFAP3 protein is the element within the motor complex that binds to the vesicle cargo [103, 104]. Defects in the KIF3 complex can lead to neurodegenerative diseases that are caused by problems with long-distance vesicle trafficking [105, 106].

With regard to synaptic plasma membrane proteins (Category 4), Caherin-15 (CDH15) was divergently expressed between populations. Cadherins are calcium-dependent cell adhesion proteins occurring on both pre- and postsynaptic membranes that align the active zone to the postsynaptic membrane, thus enabling efficient neurotransmission [107, 108]. These proteins regulate dendritic and synaptic architecture and influence function and plasticity in neurons [109–111]. Cadherins have been associated with various neural disorders, for example, deletion of CDH15 results in mental retardation [112, 113]. These studies underscore the vital roles of cadherins in synapse stabilization and gene regulation during neurotransmission [109]. For the final category, proteins that reversibly associate with plasma membrane proteins (Category 5), we did not recover any differentially-expressed genes (Table 1).

### Genetic basis of acoustic mating behaviors in other taxa

The genetic architecture of mating behaviors is understudied in anuran amphibians, with examples being largely limited to visual signals [114, 115]. Information regarding the genetic basis of acoustic signaling and processing in frogs is even more scarce [116]. Nonetheless, insight can be gained from insects that exhibit temporal variation in elements of acoustic mating signals in a similar way to many frogs. This work has shown that temporal variation has a polygenic genetic architecture across diverse insect groups [117–127]. Studies in *Drosophila melanogaster* found that candidate genes controlling pulse rate of acoustic signals include ion channel genes, transcription factors, and regulators of transcription and translation [128–131]. Quantitative trait mapping studies on Hawaiian crickets (genus *Laupala*) have shown that candidate genes underlying temporal information in acoustic signals include genes encoding a cyclic nucleotide-gated ion channel, a calcium release-activated calcium channel, a signal peptide peptidase-like protein gene, a putative synaptic vesicle related protein [127], three of which fall within the bounds of synaptic transmission gene categories targeted in this study (Table 1). In frogs, [116], suggested that membrane currents of fast pulse rate neurons in the parabranchial nucleus of the brain are likely to mediate divergence of acoustic signals among *Xenopus* species and that these differences probably involve differential expression of ion channels. Though little is known about the genetic basis for species-specific acoustic differences, their ongoing work will examine gene expression differences within specific vocal and auditory neurons across this genus [116].

### Caveats

One of the limitations of our study was the lack of population replication—we studied a pair of populations representing a single shift of *P. feriarum* from allopatry into sympatry along the Apalachicola River floodplain. This experimental design limits our ability to infer general, repeatable patterns of gene expression divergence. Since we have shown previously that this species independently formed contact zones with *P. nigrita* multiple times [55], our future work will investigate whether gene expression has diverged in a similar manner across contact zones or if populations diversifying through cascade reinforcement are undergoing non-parallel genetic divergence [5–7, 10]. A second limitation is that individuals were collected directly from the field under peak mating conditions, rather than raised in a common garden setting in the laboratory. Raising animals from different population treatments under the same conditions in the laboratory would remove confounding environmental variation across geographic sites. In future work, we will remove this source of variation by rearing experimental animals in the same setting. A third limitation of this study was our focus on whole brain gene expression, since relevant shifts in expression may be most pronounced in specific areas of the brain. Although this coarse-scale approach provided the first insight into expression divergence in this species, in future work we will utilize the information gained here to predict patterns of divergence in specific brain regions where changes in neural gene expression are most likely to have occurred [60, 116]. Comparing these patterns of divergence to those seen in non-neural and non-reproductive tissues could allow us to identify expected levels of divergence due to geography and demographic factors. We expect that this fine-scale targeted approach will enable us to uncover additional genes underlying the divergence of mating behaviors in this system.

### Future directions

Systems undergoing cascade reinforcement provide an exciting opportunity to study how speciation can be accelerated by spatially-varying selection pressures on reproductive traits generated through species interactions [3, 5–7]. The diversification of populations that can result suggests that different genes may be the targets of selection in different populations. Indeed, studies of parallel phenotypic evolution under similar selection pressures have often uncovered as much or more evidence of non-parallel than parallel divergence at the genomic sequence and gene expression levels [132–135] (but see [126]). In this way, the conditions of divergent selection generated by cascade reinforcement may drive the evolution of reproductive isolation via mechanisms such as gene expression divergence [38] or evolution of structural variants [36, 37] across conspecific populations. Moreover, at the broader level of gene network evolution, different elements of the same gene networks or even different networks may diverge in replicate populations, thereby accelerating the evolution reproductive isolation [39, 42].

In the current study, we identified seven candidate genes involved in synaptic transmission that are differentially-expressed geographically. Five of these genes encode synaptic vesicle proteins. Moreover, we found two gene networks with a concentration of neurotransmission terms that have diverged between ancestral and reinforced populations. Our future work will focus on testing the repeatability of evolution with respect to gene and gene network expression and genome structure in naturally-replicated populations undergoing cascade reinforcement. Moreover, by through examining specific brain regions, we will gain insight into how the genetic architecture underlying mate choice diverges during speciation.

## Conclusions

As Upland chorus frog (*Pseudacris feriarum*) populations experience strong selection via cascade reinforcement, the genetic pathways underlying neural processing of mating behaviors are predicted to diverge. Using Illumina RNA-seq and a new reference transcriptome for this species, we show evidence that multiple candidate synaptic transmission genes have diverged in expression between ancestral and reinforced populations. Moreover, gene networks with a significant concentration of neurotransmission enrichment terms have also diverged between these populations. Overall, we found greater divergence of gene expression between ancestral and reinforced populations, particularly in females, than between the sexes. Although some elements of our study design limit the generality of our conclusions, our results are consistent with the idea that divergent selection on reproductive behaviors promotes the evolution of expression in neural processing genes. Our findings lay the groundwork for future studies in non-model organisms that integrate neurogenetics, behavior, and evolution to understand the origin of new species.

## Methods

### Reference transcriptome sequencing

Eight tissues (brain, eye, testis, liver, heart, lung, skin, and leg muscle) from two adult allopatric male *P. feriarum* (catalog numbers ECM7962 and ECM7965, Nash County, NC, USA, N 35.95999°, W 78.07131°) were utilized to generate a reference transcriptome. The frogs in this study were wild-caught under the appropriate state scientific collecting permits for Alabama and Florida and procedures followed the Florida State University’s Animal Care and Use Committee Protocol #1747. Frogs were dissected within 5 min of euthanizing via decapitation, and tissues were immediately flash-frozen in liquid nitrogen after dissection to minimize RNA degradation. Nucleic acids were extracted using a phenol-chloroform method briefly described here: Each tissue sample was homogenized in 250 μL Trizol with a pestle and was kept on dry ice throughout the procedure. Then 50 μL of phenol-chloroform (pH 5.5) were added followed by centrifugation (12,000 g × 15 min, 4 °C) for phase separation. Nucleic acids were then precipitated with isopropyl alcohol and sodium acetate, followed by centrifugation (12,000 g × 10 min, 4 °C). The pellet was washed in ethanol and remaining liquid was allowed to evaporate at room temperature. Extracted nucleic acids were then suspended in RNase-free water, flash frozen in liquid nitrogen, and stored at − 80 °C. Quality analysis of the extraction was made by quantitation via Nanodrop (Thermo Fisher Scientific), Qubit (Thermo Fisher Scientific), and Bioanalyzer 2100 (Agilent Technologies). Samples with RNA Integrity Number (RIN) lower than 7.5 were discarded from further processing.

From the extracted RNA, sequencing libraries were constructed at the Georgia Genomics and Bioinformatics Core (Athens, GA, USA) using the KAPA Stranded mRNA-Seq Kit. Poly-T magnetic beads were used to capture extracted mRNA. Heat fragmentation at 94 °C for 6 min was carried out to obtain fragments 200-300 bp in length. Synthesis of the second cDNA strand included the incorporation of dUTP for identification and posterior selective amplification of 3′ cDNA. After amplification, A-tails and Illumina Truseq LT adaptors were added to the 3′ cDNAs, which were subsequently quantitated by Qubit and Fragment Analyzer (Agilent Technologies). Sequencing on an Illumina HiSeq 2500 platform (with 150 bp paired-end reads) was carried out at Florida State University’s College of Medicine Translational Science Laboratory.

### Reference transcriptome assembly

The processing, assembly, and annotation of the reads followed a general protocol published elsewhere [136], which is briefly described here. Raw sequence files were inspected in FastQC v0.11 and MultiQC v1.7 [137, 138] to verify their quality. All reads originating from the same tissue were pooled together and trimmed with Trim Galore! v0.6 [139] using a stringency of 3 bp and a 5′ hard trim of 9 bp to remove low quality bases. Reads were not de-duplicated. Trimmed reads were passed to SortMeRNA v2.1 [140] in order to filter out rRNA sequences based on similarity with the SILVA v111 and Rfam v11.0 databases [141, 142]. Reads were inspected again with FastQC and MultiQC to ensure quality of the data [143, 144]. Normalization of the data set was performed with the insilico_read_normalization.pl script from Trinity v2.8 [145] to obtain a maximum coverage of 50X. Normalized reads from each tissue were pooled and transcripts were assembled with Trinity v2.8 using a k-mer size of 25. Assembled transcripts were then validated with BUSCO v3 [146], run on transcriptome mode against the Tetrapoda Ortholog Database v8 [147] and with cutoff e-value = 1 × 10^− 6^. Other BUSCO parameters were left as default. In addition, blastx searches (cutoff E-value = 1 × 10^− 20^) were performed using the UniProt/SwissProt v2019_04 and the *Xenopus tropicalis* protein database (downloaded May 2019) to obtain estimates of protein length coverage.

Trinotate v3.1 [148] was used to generate gene annotations of the assembled transcripts. Annotations were collected from the UniProt/SwissProt and Pfam databases as provided by Trinotate v3.1 (updated to January 2015). The entire annotation process was automated by using the autoTrinotate.pl script included in the Trinotate distribution. The script used detection of long open reading frames (“LongOrfs”) to identify candidate coding regions in TransDecoder v5.5 [145]. The reference transcripts and the predicted TransDecoder peptides were matched against the UniProt/SwissProt database using blastx and blastp searches (E-value = 1 × 10^− 5^). In addition, protein family assignments were made by hmmscan v3.2 by querying the Pfam database. The results of the blast and hmmscan searches were then loaded into an SQLite database included in Trinotate v3.1. The resulting annotations were summarized with trinotateR [149] as implemented in R v3.6 [150]. A flowchart of the reference transcriptome assembly and annotation process is shown in the Supplemental material (Additional file 10: Fig. S9).

### Brain gene expression sequencing and processing

To detect differences in brain gene expression associated with reproductive character displacement, whole brains were dissected from 20 *P. feriarum*, ten of which were obtained from a population sympatric with *P. nigrita* (Liberty County, FL, USA, N 30.10145°, W 85.09951°), and ten of which were obtained from an allopatric population (Macon County, AL, USA, N 32.44314°, W 85.65523°; Fig. 1, Additional file 1: Table S10). We refer to the allopatric site as the ancestral population and the sympatric site as reinforced population throughout this paper because our phylogeographic and population genomic analyses indicate that the historical pattern of range expansion and current direction of gene flow is from allopatric into sympatric regions [55].

Our experimental design corresponds to a single shift by *P. feriarum* from allopatry to sympatry with *P. nigrita* without replication along the Apalachicola River drainage, corresponding to one of the expansions identified by Banker et al. [55]. Although more populations could have been sampled within the immediate geographic area to increase replication, sampling independent shifts in other river drainages would not have been an appropriate approach to replication for the questions we address in this study, due to the disparate phenotypic outcomes observed [51] and divergent gene expression profiles expected [42] in other contact zones. Frogs sampled from each population represented the two sexes in a 50:50 ratio. All frogs were captured in amplexus in the field while mating and laying eggs. Pairs were separated and immediately euthanized via decapitation as described above (*Methods*—*Reference transcriptome sequencing*). Females were not acclimated to laboratory conditions because we aimed to examine brain gene expression while frogs were at the breeding peak. Following Florida State University’s Animal Care and Use Committee protocol #1747, brains were removed in the field within 1 min of decapitation, transferred to cryotubes, flash-frozen in liquid nitrogen, and then stored at − 80 °C. RNA extraction, quality analysis, and library preparation were performed as described above. Three of the samples (two allopatric males and one sympatric male) produced poor-quality libraries and were not carried forward in downstream processing analyses. Sequencing of the 17 remaining libraries was carried out on an Illumina NovaSeq 6000 platform, with a target sequencing effort of 200 million reads per sample.

After raw reads were quality checked with FastQC/MultiQC, a Bayesian approach [151] was used to remove of adapters and merge of read pairs. Low quality bases at the start of the reads were trimmed with hts_CutTrim v1.0 [152] by setting a hard trim of 10 bp on the 5′ end. After PCR duplicates were removed with hts_SuperDeduper v1.0 [152], a second quality check was performed using FastQC/MultiQC. RNA reads were mapped to our *P. feriarum* reference transcriptome by using HISAT v2.1 [137], then BAM files were produced in SAMtools v1.9 [138]. Raw read counts per reference transcript were obtained directly from the BAM files using a custom script (see *Availability of data and materials*). One sample containing fewer than 20 million post-filtered read pairs was removed from downstream analyses (see Results).

Raw read counts per gene/transcript were collated and used as input for edgeR [139]. After CPM were calculated, transcripts with average CPM < 1 across all samples were discarded from the data set [153]. Calculation of CPM was made using a prior.count = 2 in order to avoid undefined logarithms when calculating log2 CPM. To account for large differences in gene counts among libraries, normalization factors for each sample were obtained by applying a Trimmed Mean of M-values (TMM) [154]. The resulting values were visualized using multidimensional scaling in order to check for outlier samples (plotMDS function used in R). As no outlier samples were obvious (Additional file 4: Fig. S3), the downstream analyses were conducted using all 16 of the samples.

Tagwise (gene-wise) dispersions were estimated and outlier effects were reduced with the estimateDisp function in edgeR (using the robust = T option). Tagwise (gene-wise) dispersions were estimated and outlier effects were reduced with the estimateDisp function in edgeR (using the robust = T option). After calculating the Biological Coefficient of Variation (BCV, Additional file 11: Fig. S10), negative binomial generalized linear models (GLM) were fitted for each gene. A flowchart of the RNA-Seq read processing into raw counts and differential expression analyses is available in the Supplemental material (Additional file 10: Fig. S9).

### Identification of candidate gene transcripts involved in synaptic transmission

We predicted that some genes involved in synaptic transmission during the processing of acoustic information should be differentially expressed between reinforced and non-reinforced populations. We compiled a list of these genes based on Sudhof (Table 9–1 in [67]) and Luo (Table 3–1 in [68]). This list included synaptic vesicle proteins, proteins that associate with synaptic vesicles, synaptic plasma membrane proteins, and proteins that reversibly associate with plasma membrane proteins during synaptic transmission (Table 1). Because ionotropic receptors (AMPA-type and NMDA-type glutamate and GABA_A_-type receptors) have been shown experimentally to mediate processing of temporal information in anuran acoustic signals [61, 64, 65], we also included genes for these receptors (Table 1). We identified candidate transcripts in our reference transcriptome by searching for appropriate keywords (Additional file 12) in the transcriptome annotation (see *Availability of data and materials*). Some broad search terms were restricted further if many irrelevant matches were recovered in preliminary searches. These restrictions followed functional descriptions by Mignogna and D’Adamo ([155]; Rab proteins), Hirokawa et al. ([103]; kinesins), and Pfister et al. ([156]; dyneins) and are defined in Additional file 12.

### Testing candidate synaptic transmission transcripts for differential expression

We tested for significant differential expression between three pairs of samples: sympatric vs. allopatric females, sympatric vs. allopatric males, and all sympatric vs. all allopatric frogs. Using the GLM model coefficients corresponding to these three comparisons, we tested whether or not each candidate gene was differentially expressed between each comparison. Likelihood ratio tests (LRT) were carried out using the glmLRT function from edgeR. Specifically, the LRT compared a GLM including the average log-counts of two given groups as coefficients to a null GLM in which both average log-counts are equal [157]. False-discovery rate corrections were applied to tests from each comparison [158]. Genes with *q* < 0.05 in one or more comparison were regarded as differentially expressed.

### Testing for unequal expression divergence among the four groups

We evaluated the magnitude of gene expression evolution in each of the four groups and compared the magnitudes among the groups to determine if more evolution had taken place in any of the groups. Following [159], we performed the following steps: 1) we standardized the expression levels for each gene to a mean of zero and a standard deviation of one, 2) we separated the expression data into two sets, one for candidate and one for non-candidate genes, 3) we computed the average expression level for each group/gene/set combination (producing eight values for each gene), 4) we computed a distance matrix (using the *dist* function in R) for each gene and for each set representing the pairwise divergence between four groups, and 5) we estimated from each distance matrix an expression divergence tree using neighbor joining (*nj* method in R). The resulting tip branch lengths were interpreted as the relative contribution of each group to the overall expression divergence. In Additional file 5: Fig. S4, we represent for each group the relative contribution as the log_2_ ratio of the branch lengths for two groups. To test for significance, we repeated steps 3–5 for each gene, computed the average branch length difference across the genes within the set (the test statistic), and performed randomization test in which the assignment of candidate and non-candidate was shuffled among the genes before recomputing the test-statistic. The null distribution was comprised of 10,000 replicates. In order to test whether each difference was higher in the candidate than non-candidate genes, we compared the difference (candidate minus non-candidate) to a null distribution generated using the approach above. Note that zero length branches for individual genes required that differences were used instead of ratios. The *p*-values from these tests (8 tests corresponding to the group x set combinations, and 4 tests to compare the two sets) were used to compute FDR to correct for 12 total tests.

### Evaluating transcriptome-wide patterns of divergence

In order to elucidate general expression patterns in the entire transcriptome data set, we expanded our analysis to all transcripts for the following 6 comparisons: (1) all sympatric vs. allopatric frogs, (2) all female vs. male frogs, (3) sympatric vs. allopatric females, (4) sympatric vs. allopatric males, (5) male vs. female allopatric frogs, and (6) male vs. female sympatric frogs. For the last four comparisons, a group-based analysis was performed in which each sample was assigned to a group by geography and sex. As above, to ascertain whether or not each gene was differentially expressed between each comparison, likelihood ratio tests (LRT) were carried out using the glmLRT function from edgeR. Specifically, the LRT compared a GLM including the average log-counts of two given groups as coefficients to a null GLM in which both average log-counts are equal [157]. False-discovery rate corrections were applied to each comparison and genes with FDR < 0.05 [158] were regarded as differentially expressed. We used randomization tests with 10,000 null replicates to determine: (1) whether more genes are differentially expressed by geography than by sex, (2) whether more genes are differentially expressed by geography in females than in males, and (3) whether more genes are differentially expressed by sex in sympatry than in allopatry. In each case, we computed the test statistic as the ratio of the number of differentially expressed genes (e.g. nDiffExpressedGeo/nDiffExpressedSex). To compute each sample from the null distribution, we first randomly shuffled numbers between the first and second category for each gene separately (e.g., shuffled values horizontally in a table with one column for each category and one row per gene), then recomputed the test statistic using the permuted Table. *P*-values were computed as the proportion of null replicate values that were greater than or equal to the test statistic.

Based on the resulting differentially expressed genes from the four comparisons mentioned above, a multi-dimensional scaling plot (MDS) was created to verify concordance of group membership with expression profiles using the plotMDS function. Heatmaps were also generated using normalized CPM of differentially expressed genes using the Heatmap function from the ComplexHeatmap and RColorBrewer packages [160, 161]. To obtain statistical support (approximately unbiased, AU *p*-values) and improve clustering of samples in the heatmaps, a hierarchical clustering function was applied using the pvclust and dendextend packages [69, 151]. Detection of differentially-expressed genes associated with differences between sympatric and allopatric frogs was also performed by pooling all sympatric samples in a single group, and all allopatric samples in another group (comparison 1). Similarly, tests for differences between females and males was carried out (comparison 2). All analyses were performed in R and graph support was provided by the gplots package [162].

### Weighted correlation network of brain genes

A weighted correlation network analysis was performed to detect groups of genes (modules) that showed patterns of co-expression across samples, geography (allopatry or sympatry), and sex (male or female). Co-expression networks were constructed with the R package WGCNA [163] as follows: TMM-normalized raw counts were obtained from edgeR, and in order to reduce noise from low-variation transcripts as suggested elsewhere [164], only the top 10% of genes with the highest variation in CPM across samples were retained. The resulting data set was checked for missing entries and low-variation genes using WGCNA’s function goodSampleGenes. In WGNCA, networks are assumed to follow a scale-free topology fashion, a property of biological networks in which only a few genes are highly connected, resembling a power function [165]. A scale-free topology is obtained by elevating gene correlations coefficients to a power (β) that enhances the differences between weak and strong correlations. The function pickSoftThreshold was used to iteratively ascertain which β approximated a scale-free network. The procedure fits a curve between the number of connections and the frequency of those numbers of connections in the network. A threshold was set to obtain a correlation coefficient *R*^*2*^ > 0.9 for the aforementioned curve, resulting in β = 6 (Additional file 13: Fig. S11). The transformed correlations, representing the adjacency matrix, were then converted to a Topological Overlap Matrix (TOM) which describes the overall degree of connectedness of each gene to other genes.

#### Module identification—

Genes were clustered based on expression level similarity using TOM and a hierarchical clustering method implemented in the hclust function of R. Gene models were defined by cutting the resulting dendrogram at a height of 0.99 (R function cuttreeDynamic with module size ≥40 genes and deepsplit = 3), Modules were further merge by grouping highly correlated modules based on overall module expression, with modules being merged when eigengene dissimilarities were less than 0.3. The correlation between the module expression levels and the frogs’ geography or sex were also determined using the eigengenes. The resulting *p*-values were corrected for multiple tests (25 modules x two groupings = 50 tests) using the *qvalue* function in R. A threshold of α = 0.05 was used to determine significance.

#### Module analysis—

Each of the resulting modules was visualized in Cytoscape v3.7.2 [166] after filtering of low gene-gene correlations (*r <* 0.05). Pathway enrichment analyses were performed using gene annotations in the modules from Uniprot using the R package g:Profiler2 [167]. The enrichment analysis selected gene pathways enriched at an FDR < 0.1 by obtaining annotations from the Gene Ontology (GO), Kyoto Encyclopedia of Genes and Genomes (KEGG), and TRANSFAC databases [168, 169]. The background gene set was all the annotated genes in our reference transcriptome. The enrichment analyses were made based on the unfiltered modules (i.e., low correlations were not filtered out from modules).

#### Randomization tests pertaining to modules—

In order to determine if synaptic pathways were concentrated in each of the 25 modules and also overall, we conducted randomization tests. To this aim, we performed a search for each enriched pathway (Additional file 1: Table S9) in the EMBL-EBI QuickGo database (Binns et al. 2009). If the terms “synap-,” (for synaptic transmission) or “neuro-,” or “neura-” (for neurotransmission) was found in the Ancestor Chart or Child Terms list or in the name of the enriched pathway itself, we counted it as a synaptic pathway. To test for enrichment of synaptic pathways in each separate module we used (as the test statistic) the number of synaptic pathways for that module. To test if the differentially expressed modules were enriched for synaptic pathways overall, we used (as the test statistic) the total number of synaptic pathways in the differentially expressed modules.

To simulate the null distribution to evaluate these two test statistics, we shuffled the assignment of pathway to module (holding the total number of pathways per module constant) and recomputed the test statistics (10,000 null samples were simulated). Finally, to test whether the number of synaptic pathways per differentially expressed module was higher than expected, we computed as the test statistic the average number of synaptic pathways per differentially expressed module. This test statistic was evaluated by shuffling the designation of significant/non-significant across the modules (while keeping the number of synaptic pathways pathways constant). Again 10,000 null samples were simulated. The resulting *p*-values for the 25 individual modules were corrected for multiple tests using the *qvalue* function in R. A threshold of FDR < 0.05 was used to determine significance in each test.

We also conducted an additional randomization test to determine whether modules associated with geography contained significantly more synaptic pathways than modules not associated with geography. We followed the same procedure described in the previous test, but with a single test statistic being calculated: meanNSynapticGeo/meanNSynapticNonGeo. Again, α = 0.05 was used to determine significance.

## Supplementary Information

**Additional file 1: Table S1.** Number of read pairs, average sequence length, and percentage of sequences with Phred Score. **Table S2.** Coverage of assembled trancripts against proteins in the Uniprot/Swissprot and *Xenopus tropicalis* databases. **Table S3.** Sample information and statisticts of brain RNA-Seq reads used in the differential expression analysis. Please see the main text Methods for detail on read processing and quality control. **Table S4.** Differential Expression of Candidate Synaptic Transmission Genes between reinforced (sympatric) and ancestral (allopatric) populations. The 554 candidate transcripts for which expression data were availble are shown. Signifcance of likelihood ratio tests conducted were corrected for multiple tests within each of the three sets of samples: 1) both sexes combined (left section), 2) females only (center section), and 3) males only (right section). Q-values resulting from the correction were compared to alpha = 0.05 for significance (highlighed). Note that log fold change (logFC) is expressed so that positive values mean that sympatric poplations are overexpressed relative to allopatry, whereas negative values mean that sympatric populations are underexpressed relative to allopatry. **Table S5.** Results of randomization tests comparing divergence evolution. The test statistic for the first eight tests is the difference between the branch lengths for the 2 compared groups, averaged across the genes in the set. Qvalues were obtained by correcting the *p*-values for twelve tests. **Table S6.** Differential Expression of all Transcripts between reinforced (sympatric) and ancestral (allopatric) populations (left three sections), as well as between males and females (right three sections). Signifcance of likelihood ratio tests conducted were corrected for multiple tests within each of the six sets of samples. FDR values resulting from the correction were compared to alpha = 0.05 for significance (FDR shown for significant tests only). Note that log fold change (logFC) is expressed so that positive values mean that sympatric populations are overexpressed relative to allopatry, whereas negative values mean that sympatric populations are underexpressed relative to allopatry. Note LogFC is the effect size, with positive values corresponding to overexpression in sympatry. **Table S7.** Identity, gene ontology (GO) pathways, and co-expression network module membership of differentially expressed genes. **Table S8.** Module properties and hub genes. The hub genes were selected via the chooseTopHubInEachModule function in WGCNA. The secondary annotated hub gene was defined as the gene, other than the hub gene, with the highest degree (number of connections) that included annotation. Neighborhood connectivity of a node refers to the average connectivity of all its neighbors. The clustering coefficient of a node is the ratio of connections of its neighbors and the maximum number of possible connections between neighbors. The number of differentially expressed genes bewteen any comparison is shown for each module, as well as the number of possible social/mate preference, synatic plasticity (SPG), and immediate early genes (IEG). **Table S9.** Trait-Module correlation analysis and enriched pathways (based on the *Xenopus tropicalis* and *Homo sapiens* Gene Ontology databases) at FDR < 0.1 via g:Profiler2. Signicance of correlation with Geography and Sex were corrected simultaneously (50 tests) to produce q-values. Pathway terms containing keywords (“synap-” in red and “neuro-” and “neura-” in green in Enriched pathways column) were analyzed to determine whether they were concentrated in each module (see Methods). Significance values, based on randomization tests (see Methods), for these 25 tests were corrected for multiple tests to procude q-values that were compared to 0.05 to determine significance. **Table S10.** Information of the samples used in the differential gene expression analyses. The RIN (RNA Integrity Number) are provided as measured in a Agilent Bioanalyzer. Ratios are showed as measured from Qubit (Thermo Fisher).

**Additional file 2: Figure S1.** The number of transcripts from the reference transcriptome mapping to each Gene Ontology category after gene annotation with Trinotate.

**Additional file 3: Figure S2.** Number of reads post filter for the 17 samples for which data were collected. The bottom-most sample was excluded from downstream analyses because it produced substantially fewer reads than the other 16 samples. Each bar is labeled with the corresponding Sample IDs from Additional file 1: Table S10.

**Additional file 4: Figure S3.** Multidimensional scaling (MDS) plot of pairwise expression differences, based on normalized log_2_ counts per million for genes in the transcriptome-wide gene set. This plot was used to verify that no outliers existed. Numbers inside points correspond to the last two digits of the sample IDs in Additional file 1: Table S10.

**Additional file 5: Figure S4.** A comparison of expression evolution. Tip branch lengths on the two neighbor joining trees at the top represent the amount of expression evolution in each of the four groups compared: sympatric males (SM), allopatric males (AM), sympatric females (SF), and allopatric females (AF). The bar graph below indicates the ratios of branch lengths used to compare the relative expression evolution between two groups. Error bars show the 95% bootstrap confidence interval for each comparison. Additional file 1: Table S5 presents results of randomization tests used to determine if each ratio was significantly different from zero and if ratios for candidate loci were significantly greater than corresponding ratios from non-candidate loci. None of the tests were significant at the 0.05 level after correcting for multiple tests.

**Additional file 6: Figure S5.** Relative expression levels (log_2_ CPM) of 517 differentially expressed genes between sympatric and allopatric frogs. The dendrograms resulted from hierarchical clustering of expression levels after 100 replicates to estimate Approximately Unbiased *p*-values (numbers on nodes). Gene names are Uniprot identifiers and can be found in the reference transcriptome annotation file (see *Availability of data and materials*).

**Additional file 7: Figure S6.** Relative expression levels (log_2_ CPM) of 129 differentially expressed genes between female and male frogs. The dendrograms resulted from hierarchical clustering of expression levels after 100 replicates to estimate Approximately Unbiased *p*-values (numbers on nodes). Gene names are Uniprot identifiers and can be found in the reference transcriptome annotation file (see *Availability of data and materials*).

**Additional file 8: Figure S7.** Relative expression levels (log_2_ CPM) across all samples of 24 differentially expressed genes between females and males in allopatry (A), and 34 differentially expressed genes between females and males in sympatry (B). The dendrograms resulted from hierarchical clustering of expression levels after 100 replicates to estimate Approximately Unbiased *p*-values (numbers on nodes). Gene names are Uniprot identifiers and can be found in the reference transcriptome annotation file (see *Availability of data and materials*).

**Additional file 9: Figure S8.** Correlations of geography (left column; allopatry/sympatry) or sex (right column; male/female) with each of the module’s eigengenes (rows). The color of the cells indicates positive (orange) or negative (blue) correlations. The numbers in the cell are Pearson’s coefficients with associated FDR in parentheses, which were computed by correcting *p*-values for the 50 tests. Significantly correlated traits are shown in bold and italics.

**Additional file 10: Figure S9.** Flowchart describing computational steps to process, annotate, assemble RNA-Seq reads for the reference transcriptome (green boxes), and steps to process and count reads of brain RNA-Seq for analysis of differential gene expression analysis of candidate transcripts (orange boxes) and all transcripts (blue boxes).

**Additional file 11: Figure S10.** Biological Coefficient of Variation (BCV) for each transcript in the data set. Each dot represents the average variation in transcript counts among samples (tagwise dispersion). The baseline reference used to adjust tagwise variation is indicated by dark gray line, showing the common dispersion. The model used to adjust the tagwise variation yielded a trend variation (light gray line).

**Additional file 12.** List of keywords used in the detection of synaptic transmission genes within the reference transcriptome. The list was created by querying the existing literature (see Methods-Identification of candidate genes transcripts involved in synaptic transmission).

**Additional file 13: Figure S11.** The effect of raising node correlations in the WGCNA analysis to a power β (soft threshold) on the fit (*R*^*2*^) to the assumption of a scale-free topology network. In a scale-free topology network, some nodes are highly connected (hub genes). The red line indicates a fit of *R*^*2*^ = 0.9 to a scale-free topology, indicating that gene-correlations should be elevated to a β = 6.

## Data Availability

Raw reads used to draw conclusions in this article are stored in the Sequence Read Archive (NCBI-SRA BioProject PRJNA723357). The reference transcriptome, assembled contigs, and gene annotations are stored in the Zenodo repository under Digital Object Identifier (DOI): 10.5281/zenodo.4709988 (https://zenodo.org/record/4709988#.YR7a3pNKhaw). Scripts and input files, including the matrix with read raw counts per sample are stored at Github (https://github.com/oospina/brain_gene_expr_P_feriarum). Databases were obtained from the following sources: SILVA v111 (https://www.arb-silva.de/no_cache/download/archive/release_111); Rfam v11.0 (http://ftp.ebi.ac.uk/pub/databases/Rfam/11.0); Tetrapoda Ortholog Database (OrthoDB) v8 (https://www.orthodb.org/v8/index.html); Uniprot (*Xenopus tropicalis*, https://www.uniprot.org/proteomes/UP000008143); EMBL-EBI QuickGo (www.ebi.ac.uk/QuickGO/).

## Abbreviations

ADP: Adenosine diphosphate
AMPA: α-amino-3-hydroxy-5-methyl-4-isoxazolepropionic acid
AU: Approximately unbiased *p*-value
BCV: Biological coefficient of variation
BUSCO: Benchmarking Universal Single-Copy Orthologs
cADPR: Cyclic ADP ribose
CPM: Counts per million
DE: Differentially expressed gene
FDR: False-discovery rate
F_ST_: Fixation index
GABA: Gamma aminobutyric acid
GAT: GABA transporter
GLM: Generalized linear model
GO: Gene ontology
IEG: Immediate early gene
KEGG: Kyoto Encyclopedia of Genes and Genomes
LRT: Likelihood ratio tests
LTD: Long-term depression
LTP: Long-term potentiation
MDS: Multi-dimensional scaling
NADH: Nicotinamide adenine dinucleotide
NC: Neighborhood connectivity
N_E_: Effective population size
NMDAr: N-methyl-D-aspartate receptor
NO: Nitric oxide
ORF: Open reading frame
RIN: RNA integrity number
SGN: Spiral ganglion neuron
SLC: Solute carrier
SNARE: SNAP receptor
SPG: Synaptic plasticity gene
TE: Transposable element
TMM: Trimmed mean of M-values
TOM: Topological overlap matrix
WGCNA: Weighted correlation network analysis
