# Supplementary figures and images for: Neurogenomic divergence during speciation by reinforcement of mating behaviors in chorus frogs (Pseudacris)
Source: BMC Genomics. 2021 Oct 2;22:711. doi: 10.1186/s12864-021-07995-3 (PMC8487493; doi:10.1186/s12864-021-07995-3)

**A**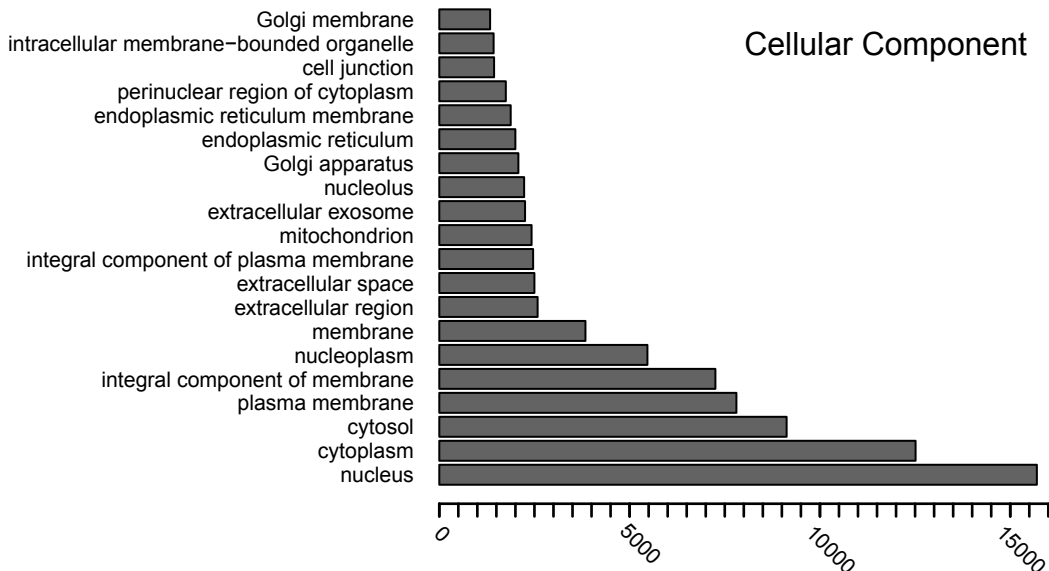**B**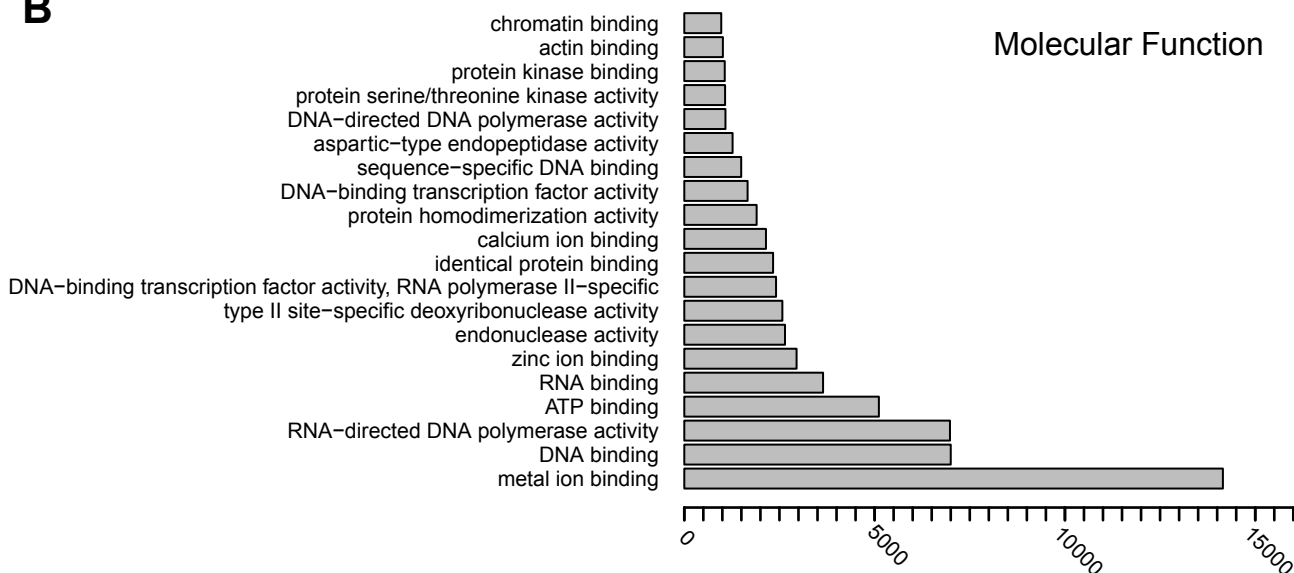**C**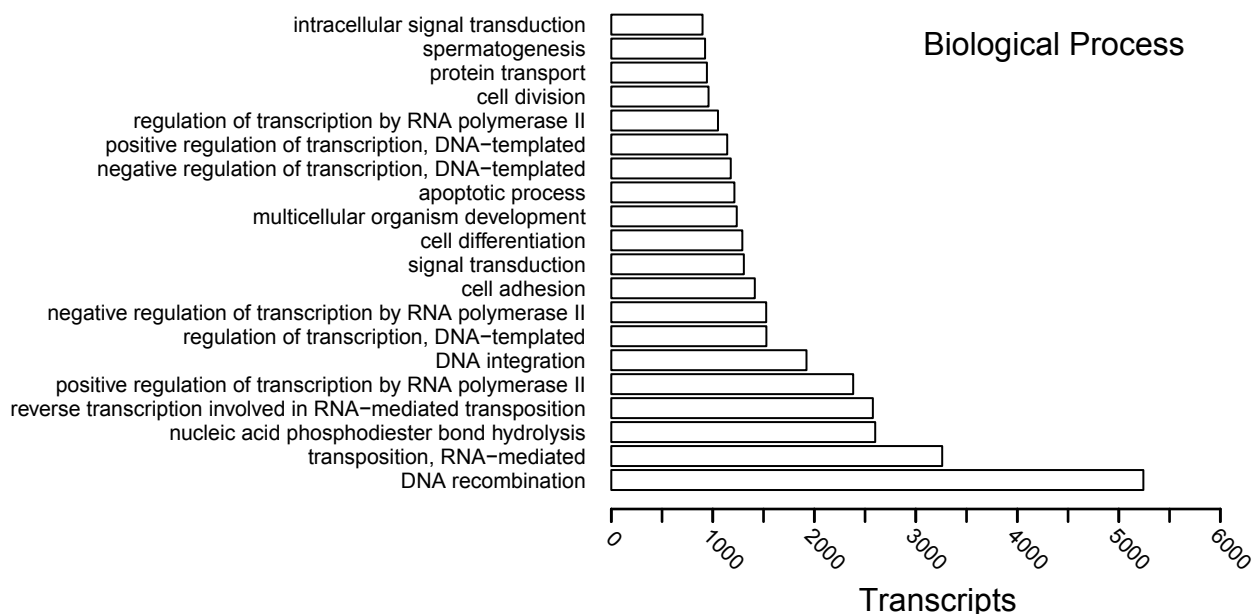

Supplement: Supplementary file 2 — Additional file 2: Figure S1. The number of transcripts from the reference transcriptome mapping to each Gene Ontology category after gene annotation with Trinotate. [file 12864_2021_7995_MOESM2_ESM.pdf]

Sample

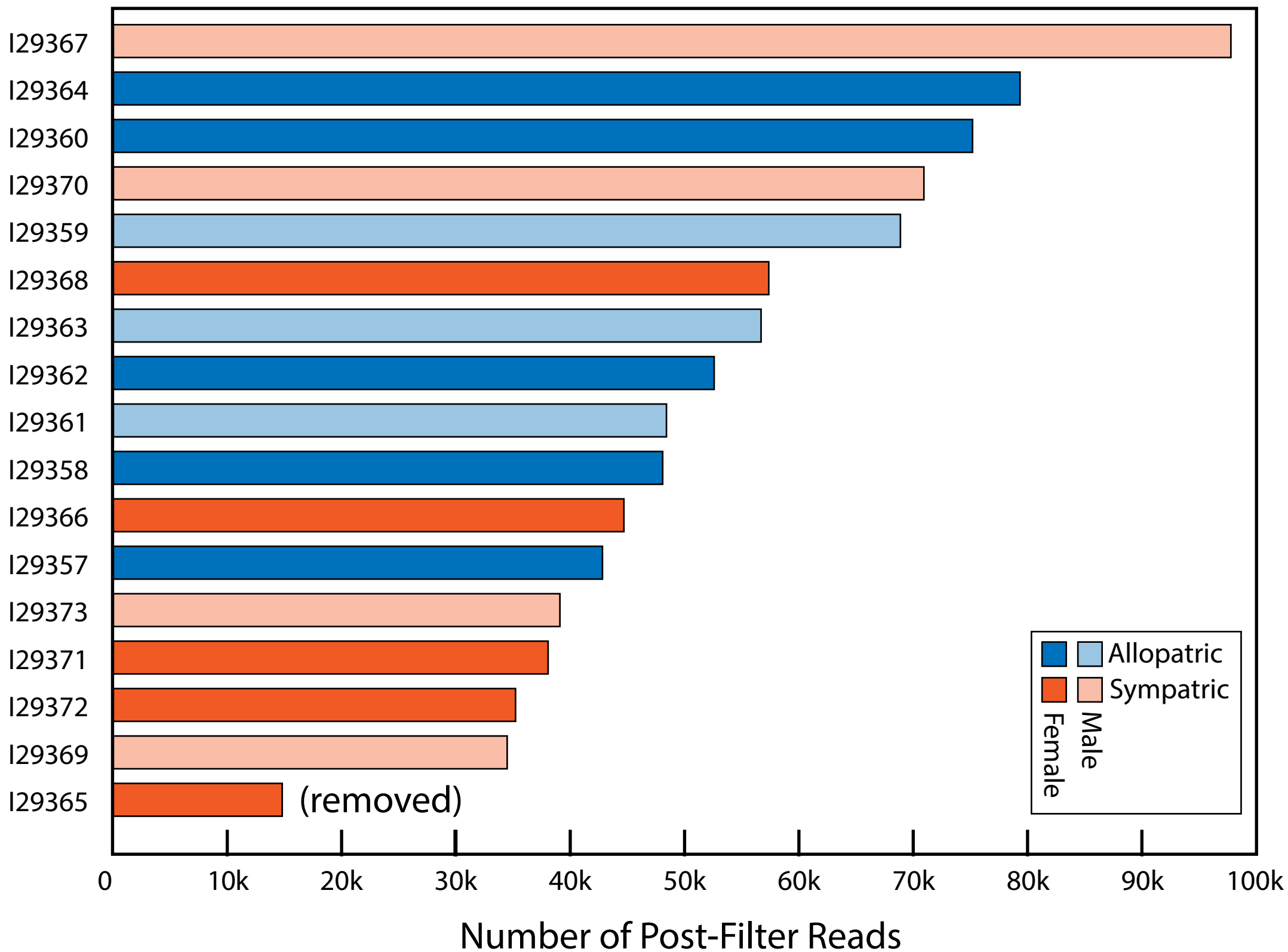

Supplement: Supplementary file 3 — Additional file 3: Figure S2. Number of reads post filter for the 17 samples for which data were collected. The bottom-most sample was excluded from downstream analyses because it produced substantially fewer reads than the other 16 samples. Each bar is labeled with the corresponding Sample IDs from Additional file 1: Table S10. [file 12864_2021_7995_MOESM3_ESM.pdf]

MDS Axis 2

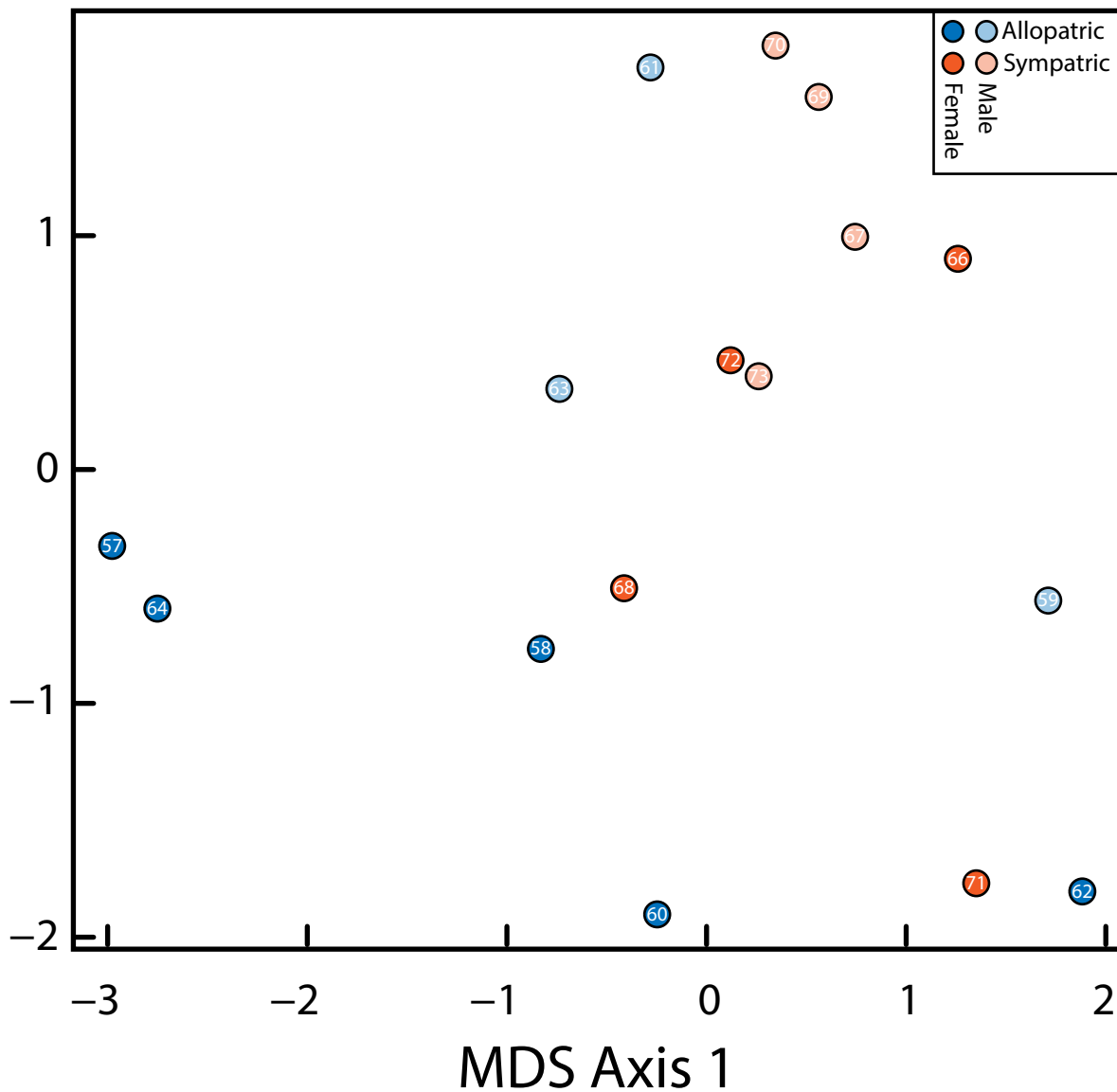

Supplement: Supplementary file 4 — Additional file 4: Figure S3. Multidimensional scaling (MDS) plot of pairwise expression differences, based on normalized log2 counts per million for genes in the transcriptome-wide gene set. This plot was used to verify that no outliers existed. Numbers inside points correspond to the last two digits of the sample IDs in Additional file 1: Table S10. [file 12864_2021_7995_MOESM4_ESM.pdf]

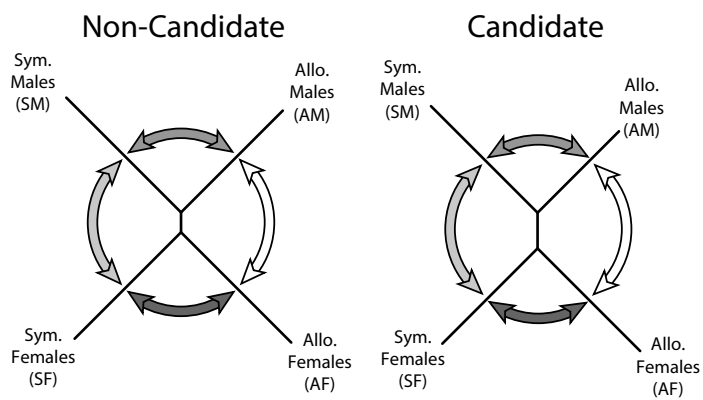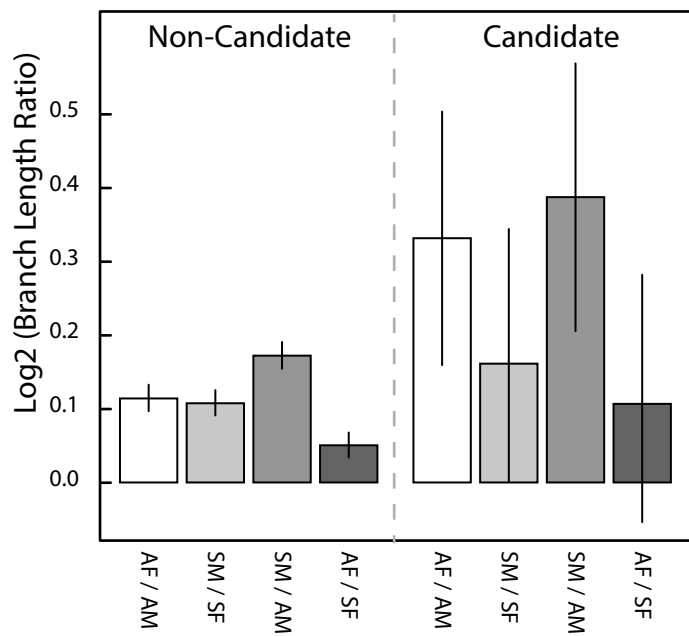

Supplement: Supplementary file 5 — Additional file 5: Figure S4. A comparison of expression evolution. Tip branch lengths on the two neighbor joining trees at the top represent the amount of expression evolution in each of the four groups compared: sympatric males (SM), allopatric males (AM), sympatric females (SF), and allopatric females (AF). The bar graph below indicates the ratios of branch lengths used to compare the relative expression evolution between two groups. Error bars show the 95% bootstrap confidence interval for each comparison. Additional file 1: Table S5 presents results of randomization tests used to determine if each ratio was significantly different from zero and if ratios for candidate loci were significantly greater than corresponding ratios from non-candidate loci. None of the tests were significant at the 0.05 level after correcting for multiple tests. [file 12864_2021_7995_MOESM5_ESM.pdf]

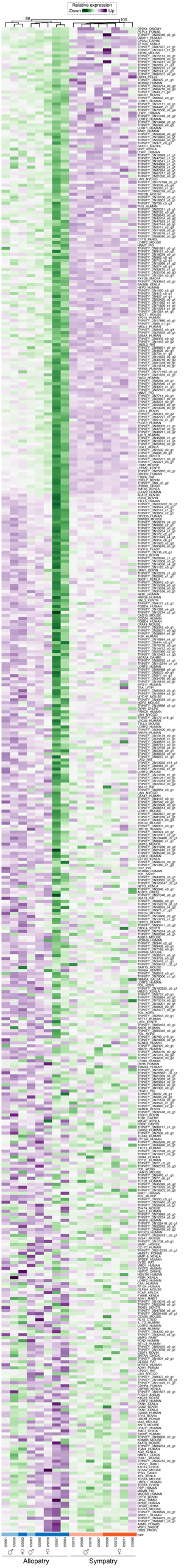

Supplement: Supplementary file 6 — Additional file 6: Figure S5. Relative expression levels (log2 CPM) of 517 differentially expressed genes between sympatric and allopatric frogs. The dendrograms resulted from hierarchical clustering of expression levels after 100 replicates to estimate Approximately Unbiased p-values (numbers on nodes). Gene names are Uniprot identifiers and can be found in the reference transcriptome annotation file (see Availability of data and materials). [file 12864_2021_7995_MOESM6_ESM.pdf]

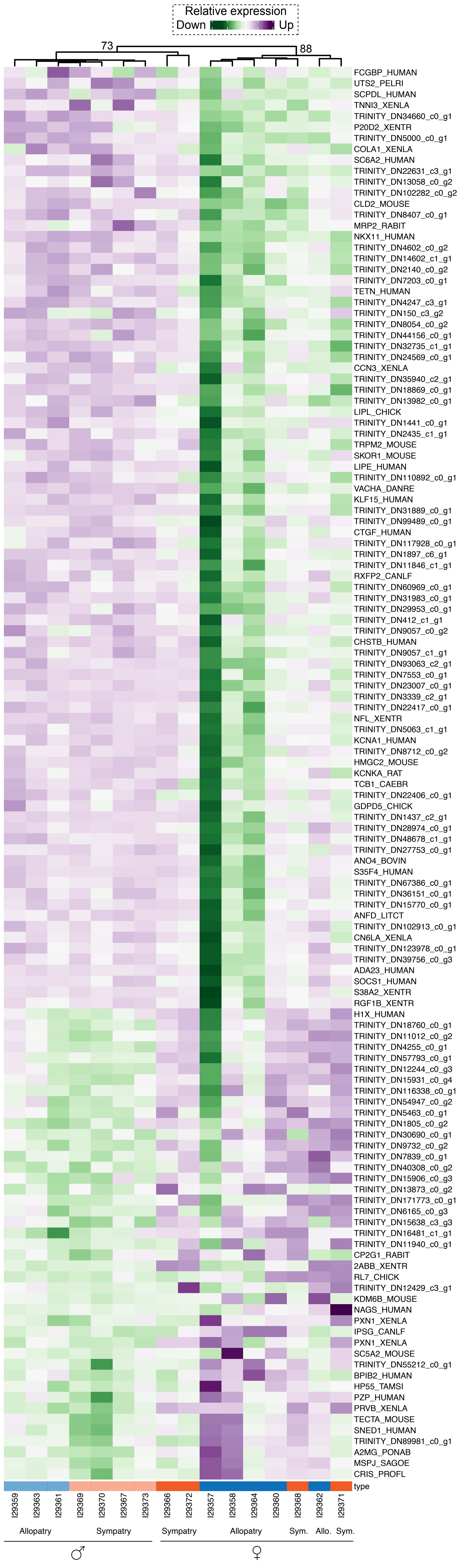

Supplement: Supplementary file 7 — Additional file 7: Figure S6. Relative expression levels (log2 CPM) of 129 differentially expressed genes between female and male frogs. The dendrograms resulted from hierarchical clustering of expression levels after 100 replicates to estimate Approximately Unbiased p-values (numbers on nodes). Gene names are Uniprot identifiers and can be found in the reference transcriptome annotation file (see Availability of data and materials). [file 12864_2021_7995_MOESM7_ESM.pdf]

**A**

Relative expression  
Down Up

**B**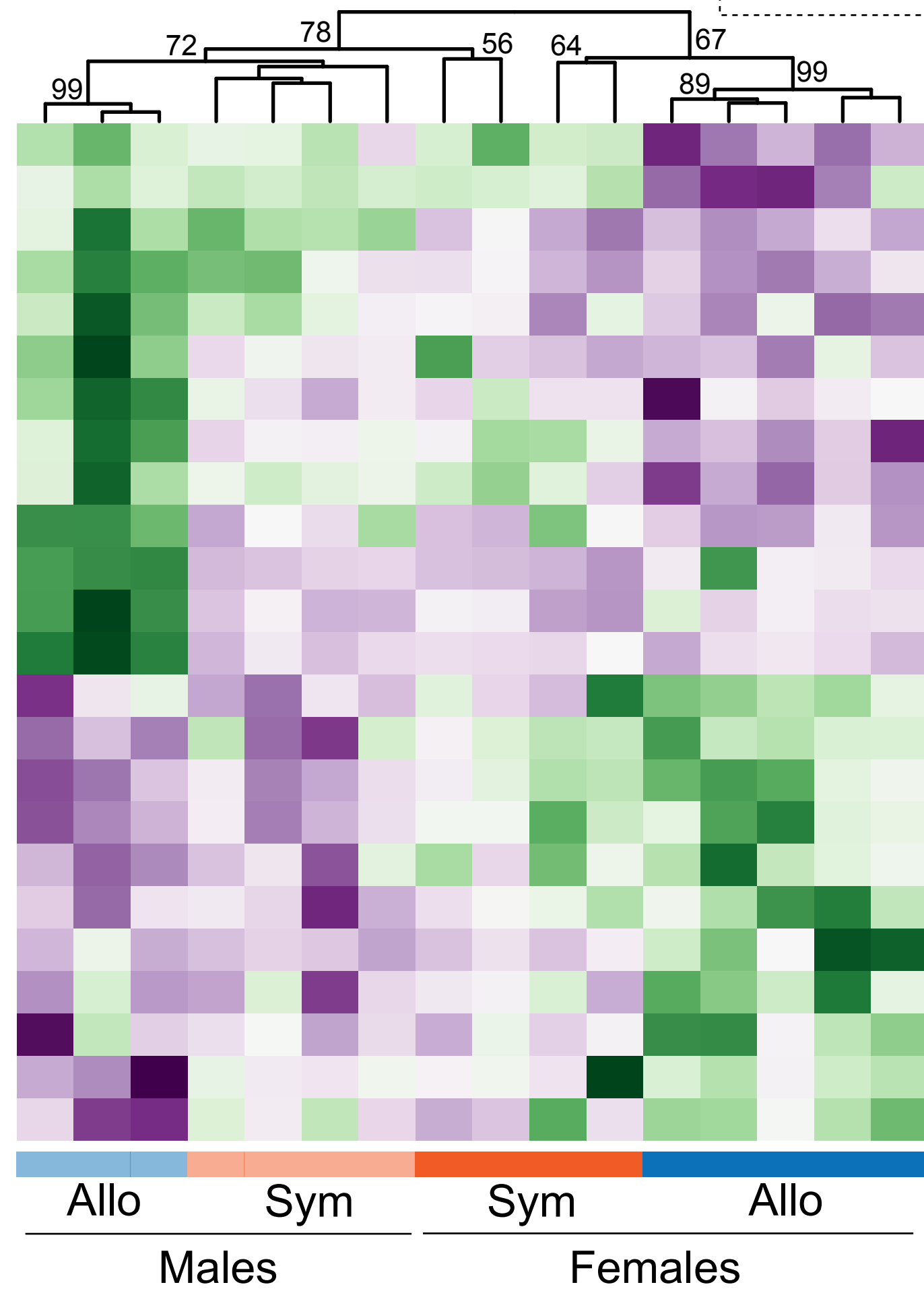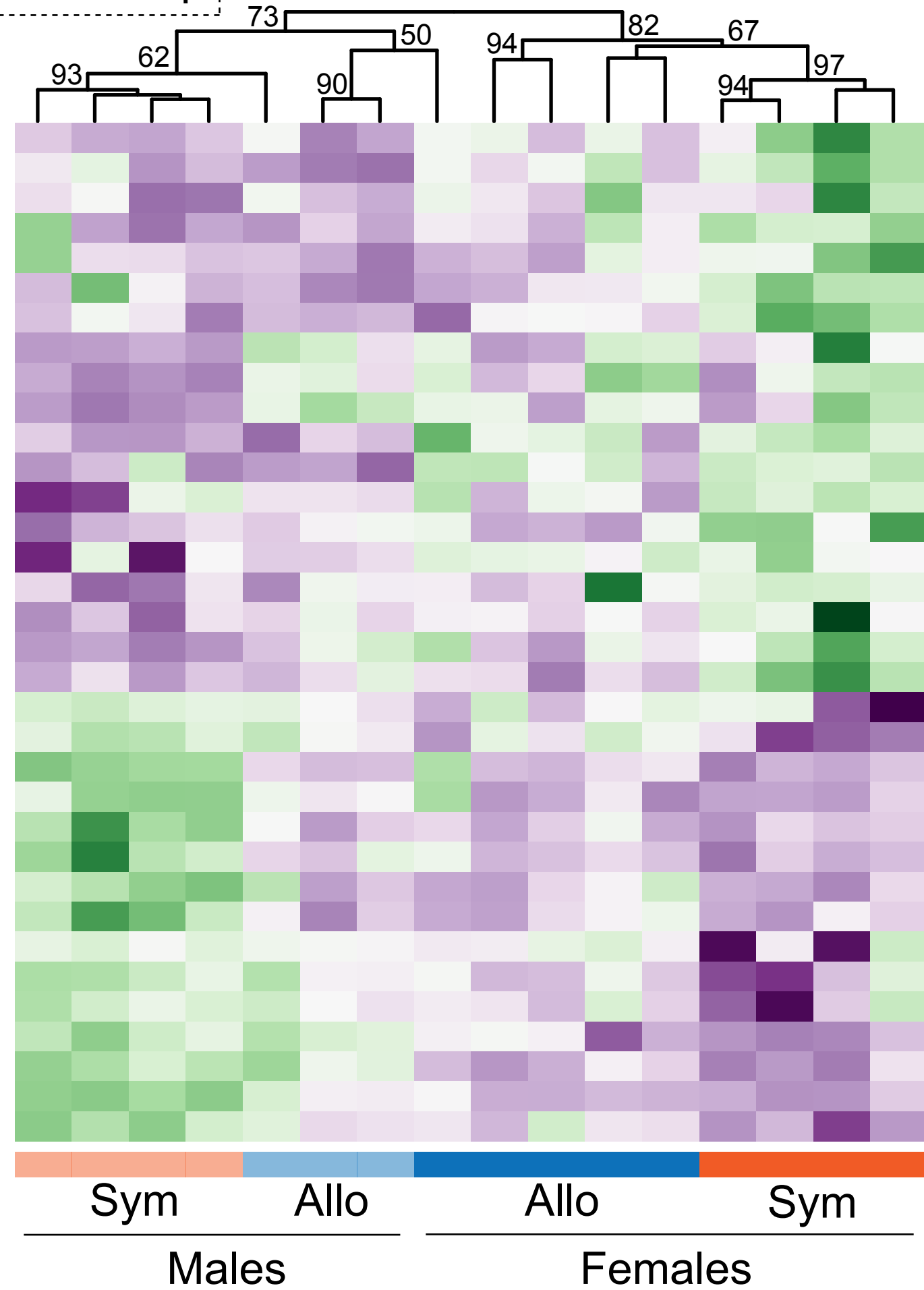

Supplement: Supplementary file 8 — Additional file 8: Figure S7. Relative expression levels (log2 CPM) across all samples of 24 differentially expressed genes between females and males in allopatry (A), and 34 differentially expressed genes between females and males in sympatry (B). The dendrograms resulted from hierarchical clustering of expression levels after 100 replicates to estimate Approximately Unbiased p-values (numbers on nodes). Gene names are Uniprot identifiers and can be found in the reference transcriptome annotation file (see Availability of data and materials). [file 12864_2021_7995_MOESM8_ESM.pdf]

Module–trait relationships

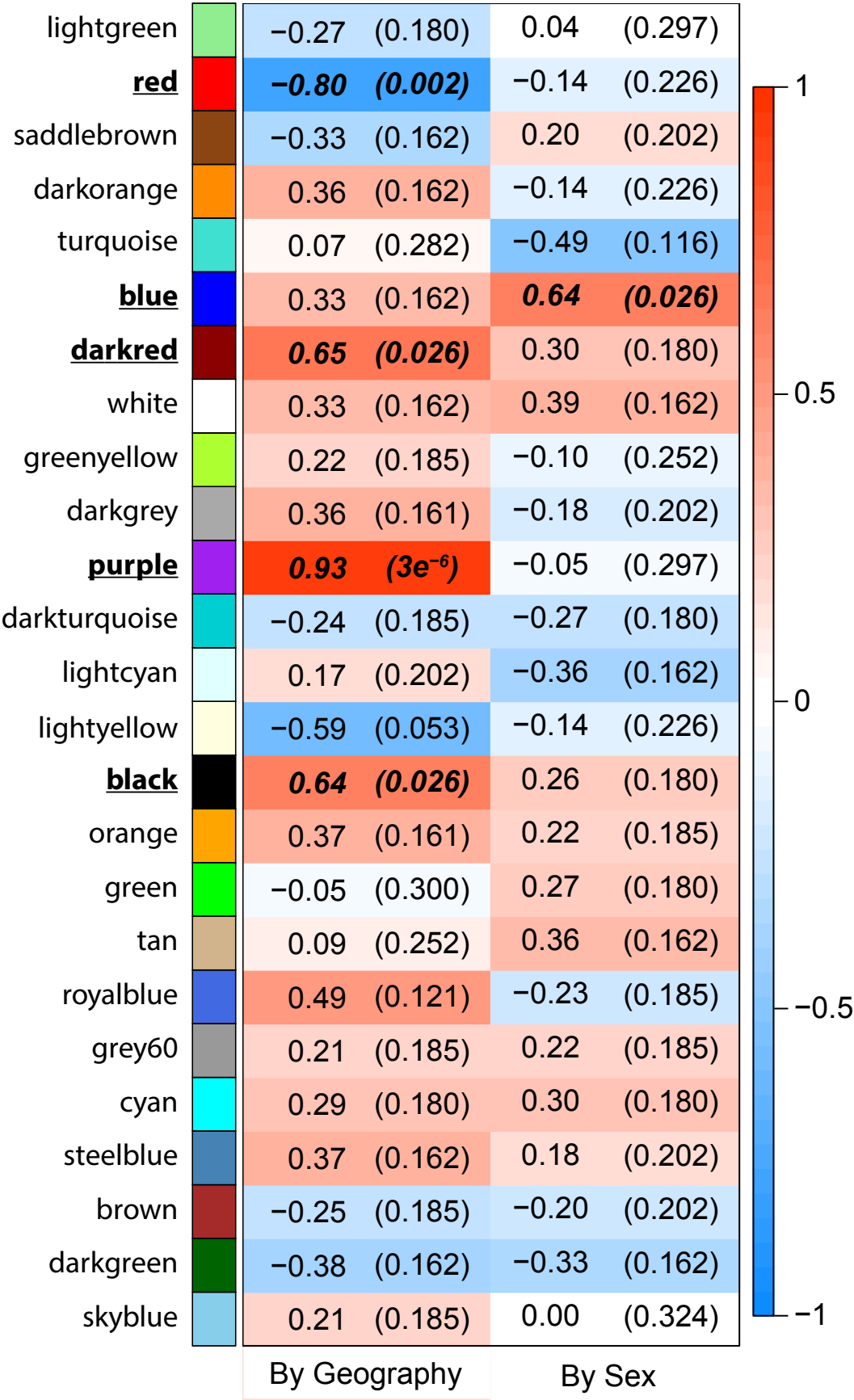

Supplement: Supplementary file 9 — Additional file 9: Figure S8. Correlations of geography (left column; allopatry/sympatry) or sex (right column; male/female) with each of the module’s eigengenes (rows). The color of the cells indicates positive (orange) or negative (blue) correlations. The numbers in the cell are Pearson’s coefficients with associated FDR in parentheses, which were computed by correcting p-values for the 50 tests. Significantly correlated traits are shown in bold and italics. [file 12864_2021_7995_MOESM9_ESM.pdf]

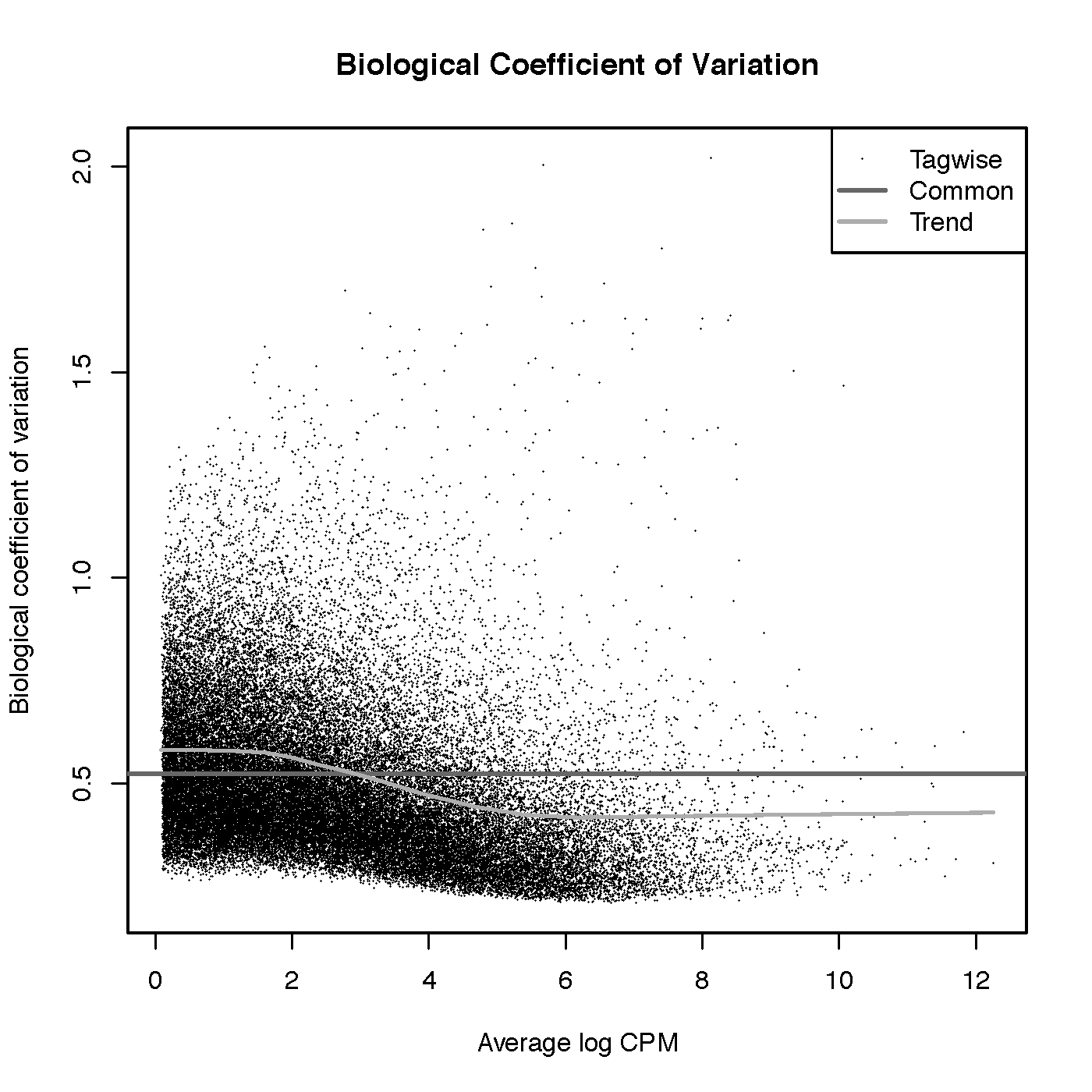

Supplement: Supplementary file 11 — Additional file 11: Figure S10. Biological Coefficient of Variation (BCV) for each transcript in the data set. Each dot represents the average variation in transcript counts among samples (tagwise dispersion). The baseline reference used to adjust tagwise variation is indicated by dark gray line, showing the common dispersion. The model used to adjust the tagwise variation yielded a trend variation (light gray line). [file 12864_2021_7995_MOESM11_ESM.tiff]

Scale-Independence of Network

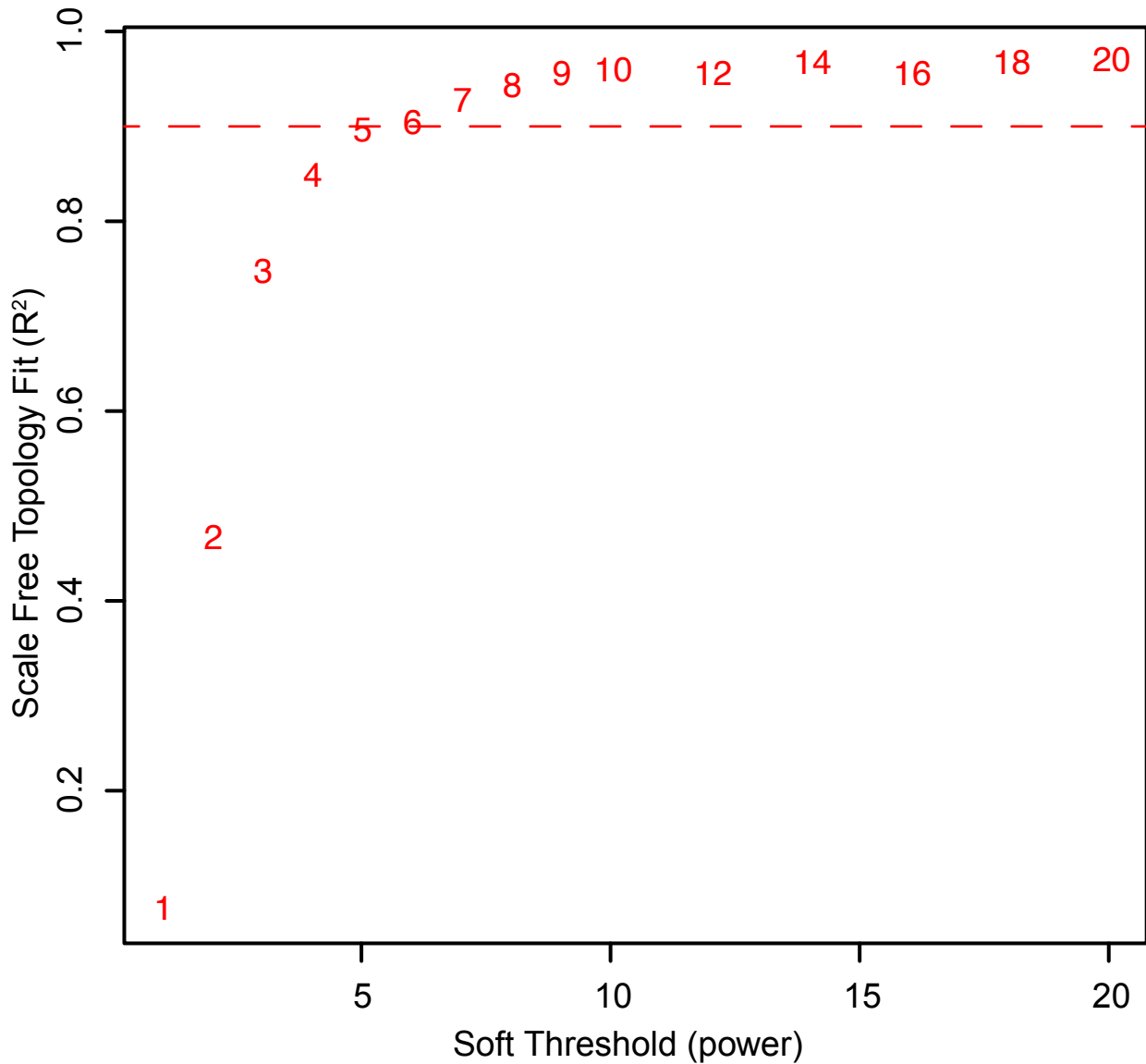

Supplement: Supplementary file 13 — Additional file 13: Figure S11. The effect of raising node correlations in the WGCNA analysis to a power β (soft threshold) on the fit (R2) to the assumption of a scale-free topology network. In a scale-free topology network, some nodes are highly connected (hub genes). The red line indicates a fit of R2 = 0.9 to a scale-free topology, indicating that gene-correlations should be elevated to a β = 6. [file 12864_2021_7995_MOESM13_ESM.pdf]
