# Supplementary material for: Neurogenomic divergence during speciation by reinforcement of mating behaviors in chorus frogs (Pseudacris)
Source: BMC Genomics. 2021 Oct 2;22:711. doi: 10.1186/s12864-021-07995-3 (PMC8487493; doi:10.1186/s12864-021-07995-3)
Supplement: Supplementary file 10 — Additional file 10: Figure S9. Flowchart describing computational steps to process, annotate, assemble RNA-Seq reads for the reference transcriptome (green boxes), and steps to process and count reads of brain RNA-Seq for analysis of differential gene expression analysis of candidate transcripts (orange boxes) and all transcripts (blue boxes). [file 12864_2021_7995_MOESM10_ESM.pdf]

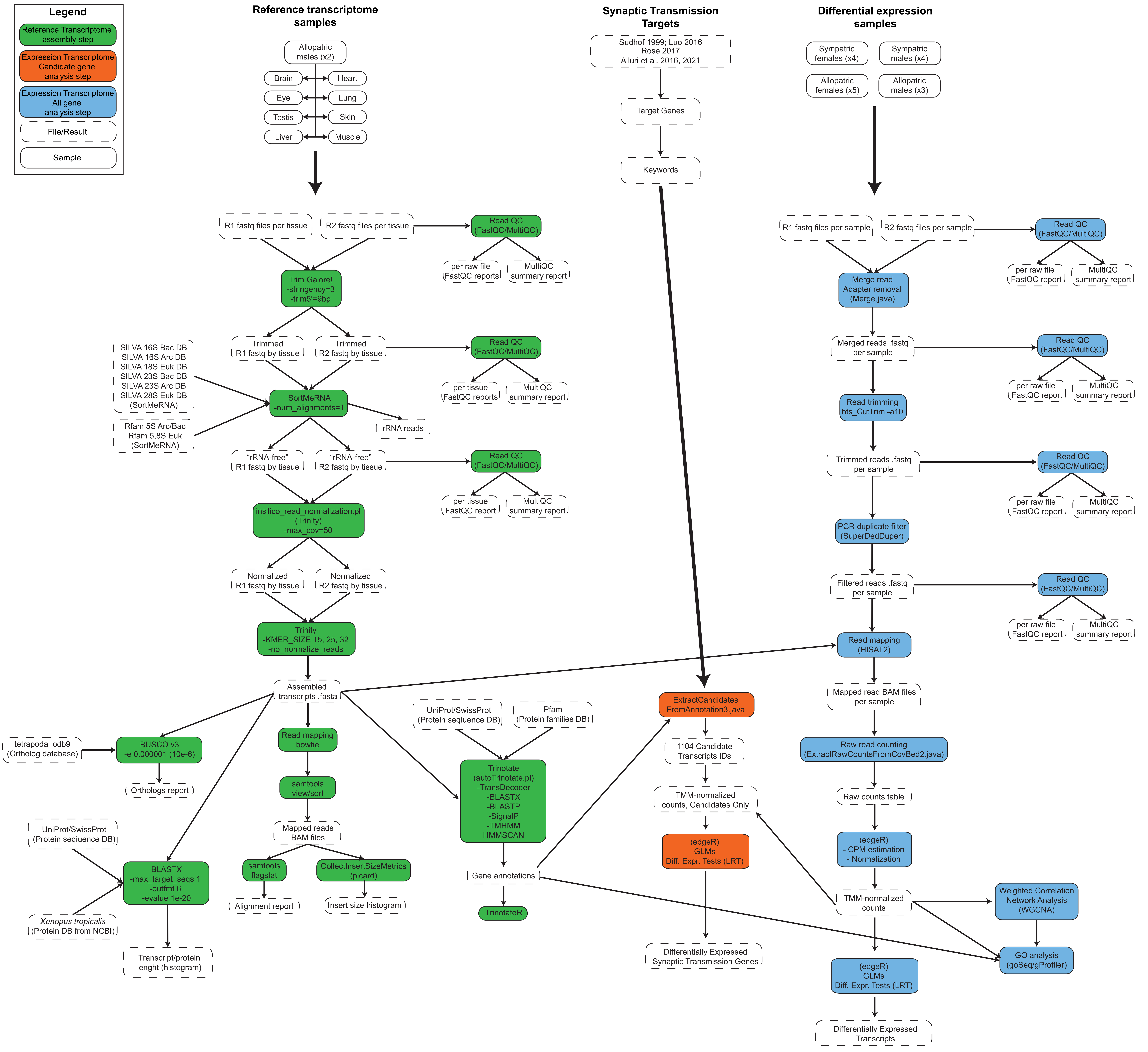

Legend
Reference Transcriptome assembly step
Expression Transcriptome Candidate gene analysis step
Expression Transcriptome All gene analysis step
File/Result
Sample
Reference transcriptome samples
Allopatric males (x2)
Brain
Heart
Eye
Lung
Testis
Skin
Liver
Muscle
R1 fastq files per tissue
R2 fastq files per tissue
Read QC (FastQC/MultiQC)
per raw file FastQC reports
MultiQC summary report
Trim Galore! -stringency=3 -trim5'=9bp
Trimmed R1 fastq by tissue
Trimmed R2 fastq by tissue
Read QC (FastQC/MultiQC)
per tissue FastQC reports
MultiQC summary report
SILVA 16S Bac DB
SILVA 16S Arc DB
SILVA 18S Euk DB
SILVA 23S Bac DB
SILVA 23S Arc DB
SILVA 28S Euk DB
(SortMeRNA)
Rfam 5S Arc/Bac
Rfam 5.8S Euk
(SortMeRNA)
SortMeRNA -num_alignments=1
rRNA reads
"rRNA-free" R1 fastq by tissue
"rRNA-free" R2 fastq by tissue
Read QC (FastQC/MultiQC)
per tissue FastQC report
MultiQC summary report
insilico_read_normalization.pl (Trinity) -max_cov=50
Normalized R1 fastq by tissue
Normalized R2 fastq by tissue
Trinity -KMER_SIZE 15, 25, 32 -no_normalize_reads
Assembled transcripts .fasta
tetrapoda_odb9 (Ortholog database)
BUSCO v3 -e 0.000001 (10e-6)
Orthologs report
UniProt/SwissProt (Protein seqiuence DB)
Xenopus tropicalis (Protein DB from NCBI)
BLASTX -max_target_seqs 1 -outfmt 6 -evalue 1e-20
Transcript/protein lenght (histogram)
Read mapping bowtie
samtools view/sort
Mapped reads BAM files
samtools flagstat
CollectInsertSizeMetrics (picard)
Alignment report
Insert size histogram
UniProt/SwissProt (Protein seqiuence DB)
Pfam (Protein families DB)
Trinotate (autoTrinotate.pl) -TransDecoder -BLASTX -BLASTP -SignalP -TMHMM HMMSCAN
Gene annotations
TrinotateR
Synaptic Transmission Targets
Sudhof 1999; Luo 2016 Rose 2017 Alluri et al. 2016, 2021
Target Genes
Keywords
ExtractCandidates FromAnnotation3.java
1104 Candidate Transcripts IDs
TMM-normalized counts, Candidates Only
(edgeR) GLMs Diff. Expr. Tests (LRT)
Differentially Expressed Synaptic Transmission Genes
Differential expression samples
Sympatric females (x4)
Sympatric males (x4)
Allopatric females (x5)
Allopatric males (x3)
R1 fastq files per sample
R2 fastq files per sample
Read QC (FastQC/MultiQC)
per raw file FastQC report
MultiQC summary report
Merge read Adapter removal (Merge.java)
Merged reads .fastq per sample
Read QC (FastQC/MultiQC)
per raw file FastQC report
MultiQC summary report
Read trimming hts_CutTrim -a10
Trimmed reads .fastq per sample
Read QC (FastQC/MultiQC)
per raw file FastQC report
MultiQC summary report
PCR duplicate filter (SuperDedDuper)
Filtered reads .fastq per sample
Read QC (FastQC/MultiQC)
per raw file FastQC report
MultiQC summary report
Read mapping (HISAT2)
Mapped read BAM files per sample
Raw read counting (ExtractRawCountsFromCovBed2.java)
Raw counts table
(edgeR) - CPM estimation - Normalization
TMM-normalized counts
Weighted Correlation Network Analysis (WGCNA)
GO analysis (goSeq/gProfiler)
(edgeR) GLMs Diff. Expr. Tests (LRT)
Differentially Expressed Transcripts
